# Dysregulation of transposable elements and PIWI-interacting RNAs in myelodysplastic neoplasms

**DOI:** 10.1186/s40364-025-00725-x

**Published:** 2025-01-23

**Authors:** Zdenek Krejcik, David Kundrat, Jiri Klema, Andrea Hrustincova, Iva Trsova, Monika Belickova, Jaroslav Cermak, Anna Jonasova, Jiri Dostal, Michaela Dostalova Merkerova

**Affiliations:** 1 Department of Genomics, Institute of Hematology and Blood Transfusion, Prague, Czech Republic; 2 Department of Computer Science, Faculty of Electrical Engineering, Czech Technical University in Prague, Prague, Czech Republic; 3 Laboratory of Anemias, Institute of Hematology and Blood Transfusion, Prague, Czech Republic; 4 First Department of Medicine, General University Hospital, Prague, Czech Republic; 5 Department of Biochemistry, Institute of Organic Chemistry and Biochemistry, Czech Academy of Sciences, Prague, Czech Republic

**Keywords:** Myelodysplastic neoplasms, Transposable elements, piRNA, Next-generation sequencing, Biomarkers, Bioinformatics

## Abstract

**Background:**

Myelodysplastic neoplasms (MDS) are heterogeneous hematopoietic disorders characterized by ineffective hematopoiesis and genome instability. Mobilization of transposable elements (TEs) is an important source of genome instability leading to oncogenesis, whereas small PIWI-interacting RNAs (piRNAs) act as cellular suppressors of TEs. However, the roles of TEs and piRNAs in MDS remain unclear.

**Methods:**

In this study, we examined TE and piRNA expression through parallel RNA and small RNA sequencing of CD34+ hematopoietic stem cells from MDS patients.

**Results:**

Comparative analysis of TE and piRNA expression between MDS and control samples revealed several significantly dysregulated molecules. However, significant differences were observed between lower-risk MDS (LR-MDS) and higher-risk MDS (HR-MDS) samples. In HR-MDS, we found an inverse correlation between decreased TE levels and increased piRNA expression and these TE and piRNA levels were significantly associated with patient outcomes. Importantly, the upregulation of *PIWIL2*, which encodes a key factor in the piRNA pathway, independently predicted poor prognosis in MDS patients, underscoring its potential as a valuable disease marker. Furthermore, pathway analysis of RNA sequencing data revealed that dysregulation of the TE‒piRNA axis is linked to the suppression of processes related to energy metabolism, the cell cycle, and the immune response, suggesting that these disruptions significantly affect cellular activity.

**Conclusions:**

Our findings demonstrate the parallel dysregulation of TEs and piRNAs in HR-MDS patients, highlighting their potential role in MDS progression and indicating that the *PIWIL2* level is a promising molecular marker for prognosis.

**Graphical Abstract:**

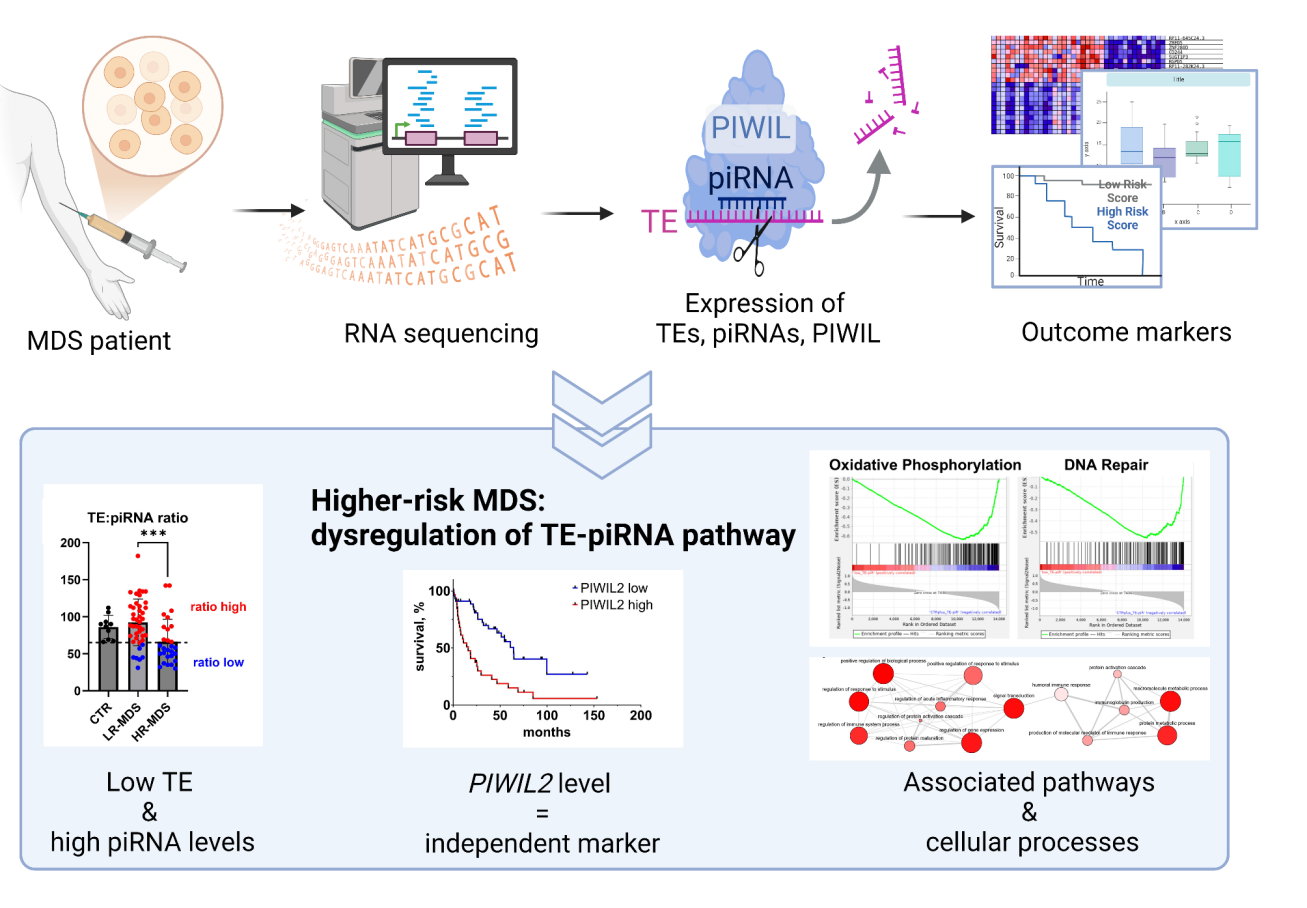

**Supplementary Information:**

The online version contains supplementary material available at 10.1186/s40364-025-00725-x.

## Introduction

Myelodysplastic neoplasms (MDS) are hematopoietic stem cell (HSC) disorders characterized by bone marrow (BM) dysplasia, ineffective hematopoiesis, and an increased risk of progression to acute myeloid leukemia (AML) [1]. MDS include a spectrum of dynamic disorders in which clonal evolution triggers disease onset and progression [2]. Given that normal HSCs give rise to all mature blood cell lineages, their ability to maintain genomic stability is essential for proper hematopoiesis and needs to be highly regulated. In MDS, an initiating mutation in HSCs leads to genomic instability, promoting the accumulation of additional somatic mutations, impaired differentiation, and eventual transformation into AML [3].

One of the major contributors to genome instability in MDS is the activation of transposable elements (TEs), which are homologous DNA sequences scattered throughout the genome [4]. TEs can cause genetic instability through homologous recombination, which may lead to their integration into gene exons, resulting in frameshifts and the production of aberrant proteins [5]. TE transcription is often elevated in cancers, posing risks to genomic stability [6]. However, recent studies revealed that TEs can have not only deleterious but also beneficial effects. The induction of TE expression leads to the activation of the viral recognition pathway and cancer cell death (immune-mediated cell clearance) [7].

The mobilization of TEs is tightly regulated by PIWI-interacting RNAs (piRNAs) [8]. These small noncoding RNAs differ from their well-known relatives, microRNAs (miRNAs), in their length (piRNAs: 26–31 nt, miRNAs: 21–23 nt), biogenesis, associated proteins, and functional roles [9]. Unlike miRNAs, which broadly fine-tune gene expression through complementarity to mRNA targets, piRNAs primarily safeguard genomic integrity in the germline by silencing TEs via interactions with PIWI proteins, which are members of the Argonaute family [10]. These PIWI-piRNA complexes silence TEs by promoting heterochromatin formation or direct transcript degradation, thereby preserving genomic integrity [8]. In addition to their role in germline cells, piRNAs and PIWI proteins have also been implicated in somatic cells, including cancer cells, suggesting that they may also contribute to genomic stability in these cells [11, 12].

Despite increasing evidence of TE and piRNA involvement in cancer [13, 14], their roles in MDS remain poorly understood. Most studies to date have focused on AML rather than MDS. For example, Colombo et al. [7, 15] published two studies on the mobilization of TEs in AML. These researchers analyzed alterations in TE expression and identified a TE expression signature that predicts prognosis in AML [15]. This group also demonstrated that the expression of TEs is significantly suppressed in leukemic stem cells of AML and higher-risk MDS (HR-MDS) patients [7]. Two studies, which focused predominantly on miRNAs, explored piRNA expression in MDS only marginally [16, 17]. Hrustincova et al. [16] sequenced circulating RNAs in plasma and reported that only a few plasma piRNAs were dysregulated in MDS. Beck et al. [17] reported piRNA enrichment in unsorted BM cells from patients with refractory anemia (RA), a lower-risk MDS (LR-MDS) subtype, and reported that *PIWIL1* and *PIWIL2* expression was significantly upregulated in RA patients compared with controls and HR-MDS patients. On the basis of these observations, two hypotheses have emerged: Colombo et al. [7] suggested that specific induction of TEs in LR-MDS could drive immune-mediated elimination of cancer cells, whereas Beck et al. [17] proposed that piRNA enrichment in LR-MDS might help protect the genome from the accumulation of mutations.

In this study, we aim to further investigate these hypotheses and explore the interplay of TEs and piRNAs in MDS and leukemogenesis. Using next-generation sequencing (NGS), we analyze the expression of TEs and piRNAs in MDS patients at different stages of the disease (a schematic flowchart summarizing our methodology is provided in SI Fig. 1). Our findings reveal that total TE expression is significantly reduced in HR-MDS patients, and this reduction is accompanied by an increase in total piRNA levels. We also show that dysregulated expression of several TE/piRNA-related molecules correlates with shorter patient survival, with high *PIWIL2* expression serving as an independent negative prognostic marker in MDS. Moreover, the dysregulation of key biological processes associated with these changes suggests that MDS cells with concomitant low levels of TEs and high levels of piRNAs undergo substantial alterations in cellular activity.

## Materials and methods

### Patients

The study included BM samples from 80 MDS patients and 17 healthy donors as controls. Samples were collected during routine clinical assessment at the Institute of Hematology and Blood Transfusion and at the General Faculty Hospital, both in Prague, Czech Republic. Written informed consent was obtained from each tested subject, and the study was approved by the Institutional Scientific Boards and the Local Ethics Committees and performed in accordance with the ethical standards of the Declaration of Helsinki. MDS diagnosis was performed on the basis of the standard 2016 WHO classification criteria [18]. Patients were divided into the LR-MDS (IPSS-R ≤ 3.5) and HR-MDS (IPSS-R ≥ 4) groups according to the Revised International Prognostic Scoring System (IPSS-R) prognostic scoring system [19], as suggested in [20]. The presence of somatic mutations in 60 genes and cytogenetic aberrations associated with myeloid malignancies was assessed at the time of diagnosis as a part of routine clinical practice. The detailed clinical and laboratory characteristics of the cohort, including follow-up information, are summarized in SI Table 1.

**Table 1:** Multivariate Cox regression analysis for the OS and PFS of MDS patients. The input data included clinical variables (blast count, hemoglobin level, counts of neutrophils and thrombocytes, and cytogenetics), age, sex, and experimental expression data (piR_018780, FAM, HERV-Fc1, and *PIWIL2*). Only the variables that remained significant after stepwise variable selection are listed and sorted in descending order of their significance. HR – hazard ratio; CI – confidence interval

Overall survival			
Variable	HR	95% CI	*p*
High age	1.067	1.029–1.107	0.0004
High *PIWIL2*	3.197	1.618–6.314	0.0008
Low platelets	3.492	1.560–7.818	0.0024
**Progression-free survival**			
**Variable**	**HR**	**95% CI**	***p***
High *PIWIL2*	3.637	1.771–7.470	0.0004
High age	1.071	1.029–1.114	0.0007
Low platelets	4.400	1.864–10.383	0.0007
Female sex	0.340	0.170–0.681	0.0023

### Cell separation and RNA extraction

Mononuclear cells (MNCs) were separated from BM aspirates by Ficoll-Hipaque density gradient centrifugation (GE Healthcare, Munich, Germany). CD34+ hematopoietic stem/progenitor cells were isolated from MNCs via magnetic cell separation (Miltenyi Biotec, Bergisch Gladbach, Germany). Total RNA was isolated via the phenol‒chloroform method. The RNA quality was evaluated with a Qubit 3 fluorometer (Thermo Fisher Scientific, Waltham, MA, USA) and an Agilent 4200 TapeStation instrument (Agilent Technologies, Santa Clara, CA, USA).

### RNA-seq

Ribosomal RNA was depleted from total RNA samples via a RiboCop rRNA depletion kit (Lexogen, Vienna, Austria). The RNA-seq libraries were constructed via the NEBNext Ultra II Directional RNA Library Prep Kit (New England Biolabs, Ipswich, MA, USA). Libraries were pair-end sequenced (2 × 100 cycles) on a NovaSeq 6000 instrument (Illumina, San Diego, CA, USA), resulting in 85 million reads per sample on average. After quality control by FastQC, adaptor trimming and low-quality sequence removal were performed via Trimmomatic. TEs were identified and counted via the SalmonTE tool [21]. Differential expression analysis (DEA) was performed after data normalization via DESeq2 via WebMeV [22]. The binary logarithm of the fold change (log_2_FC) and false discovery rate (FDR, adjusted *p* value) values were calculated. The differences were considered statistically significant when the FDR was < 0.05.

### Small RNA-seq

Small RNA-seq libraries were prepared from total RNA via the QIAseq miRNA Library Kit (Qiagen, Hilden, Germany). Libraries were subjected to 83 cycles of single-end sequencing on a NovaSeq 6000 instrument (Illumina), resulting in 35 million reads per sample on average. The data were further processed via a custom pipeline. Briefly, after quality control and adapter trimming, sequencing reads were filtered out to include only 24–34 nt inserts, mapped to the piRNABank database [23], and deduplicated via the UMI_tool [24]. Only alignments with 100% matches for both alignment and length were retained to avoid mapping bias at both levels. DEA was performed after data normalization via DESeq2 with the same outputs as those used for the RNA-seq data analysis.

### Quantification of individual transcripts

The expression of individual transcripts (piR_018780, FAM, and *PIWIL2*) was measured via real-time quantitative PCR (RT‒qPCR) or end-point droplet digital PCR (ddPCR). Reverse transcription was performed from total RNA via the TaqMan MicroRNA Reverse Transcription Kit and piRNA-specific RT primer (piR_018780 analysis) or via the SuperScript VILO cDNA Synthesis Kit with random hexamer primers (FAM and *PIWIL2* analyses). Furthermore, TE and piRNA transcripts were quantified via RT‒qPCR via custom TaqMan assays, TaqMan Universal Master Mix (all Thermo Fisher Scientific), and a StepOnePlus instrument (Life Technologies, Carlsbad, CA, USA). *PIWIL2* expression was measured via ddPCR via a standard TaqMan assay and 2X ddPCR Supermix for Probes (Bio-Rad, Hercules, CA, USA) on a Bio-Rad QX200 ddPCR system. All the target levels were normalized to the level of the reference gene *HPRT1* [25]. All the assay IDs and sequences of custom primers and probes are listed in SI Table 2.

### Statistical analysis

Statistical analyses were performed via GraphPad Prism 10 software (La Jolla, CA, USA). Student’s t test and Welch’s ANOVA were used to compare variables between groups of samples. The Pearson test was performed to assess the correlation of continuous variables. The survival distributions for overall survival (OS) and progression-free survival (PFS) were estimated via the Kaplan‒Meier method, and the differences were compared via the log-rank test. For multivariate analysis (MVA), we estimated a Cox proportional hazards regression model via MedCalc v22 software (Ostend, Belgium). Continuous values for clinical parameters were transformed to ordinal values using the standard cutoff ranges according to the IPSS-R criteria [19]. For the expression analyses, patients were sorted into high- and low-expression groups, with the expression levels detected in the CTR group as the cutoffs (details in the Results). The stepwise variable selection method was applied to retain only the independent variables significantly contributing to the predictive power of the resulting model. For the analyses, all MDS patients (or their subsets) and controls served as biological replicates. Differences were considered statistically significant when *p* < 0.05.

### Pathway analysis

The functional changes related to the dysregulation of gene expression were assessed via gene set enrichment analysis (GSEA) [26]. As a reference, hallmark (h.all.v7.0.symbols.gmt [Hallmarks]) gene sets from the Molecular Signatures Database were utilized. The enriched terms with a recommended FDR < 0.2 were further considered. The GOrilla [27] and Revigo [28] reduction and visualization tools for Gene Ontology (GO) enrichment analysis were also employed.

### Integrative data analysis

For the selected molecules, correlation networks were constructed according to normalized expression data for protein-coding genes (PCGs), TEs, and piRNAs via computational modeling as we previously described [25]. Briefly, pairwise correlations between all molecules were assessed, creating an extensive and complete undirected correlation graph. Subgraphs corresponding to each of the selected molecules were extracted, and only the most strongly correlated neighbors and the neighbors of these neighbors were included in the subgraph. All edges whose absolute correlation was higher than the 3rd quartile of correlations of the complete module graph were then visualized with the Cytoscape tool [29]. Enrichment analysis was carried out for each module with clusterProfiler [30] and was based on annotations of all the PCGs included in the corresponding module. The Revigo reduction tool was finally utilized to remove redundant GO terms.

## Results

### General characteristics of TE expression in MDS

To characterize TE expression in CD34+ BM cells from MDS patients, we performed RNA-seq analysis of samples from all 80 MDS patients and 17 controls. Overall, we identified 687 TEs across the cohort. The most abundant class of TEs was short interspersed nuclear elements (SINEs), which accounted for approximately 79% of all TEs on average. In terms of frequency, SINEs were followed by L1s (LINE1s; long interspersed nuclear elements; 15% of all TEs) and ERVs (endogenous retroviruses; ERV1, ERV2, and ERV3; together accounting for 6% of all TEs). Very low frequencies (< 1%) were observed for the other classes of TEs.

DEA revealed similar expression profiles between the MDS patients and controls, with only a few differentially expressed TEs (FDR < 0.05) [11 upregulated TEs (ERV1: HERV-Fc1, MER51E, LTR27E, MER65C, HUERS-P1, PABL_A, and LOR1I; ERV3: MER54B and LTR75; CR1: X5A_LINE, SINE: MIR3) and two downregulated TEs (ERV1: LTR71A and LTR26E)] in the MDS group (SI Fig. 2). SI Table 3 shows the list of the dysregulated TEs, including their classes, clades, normalized counts, log_2_FC values, and FDRs.

**Fig. 1:**
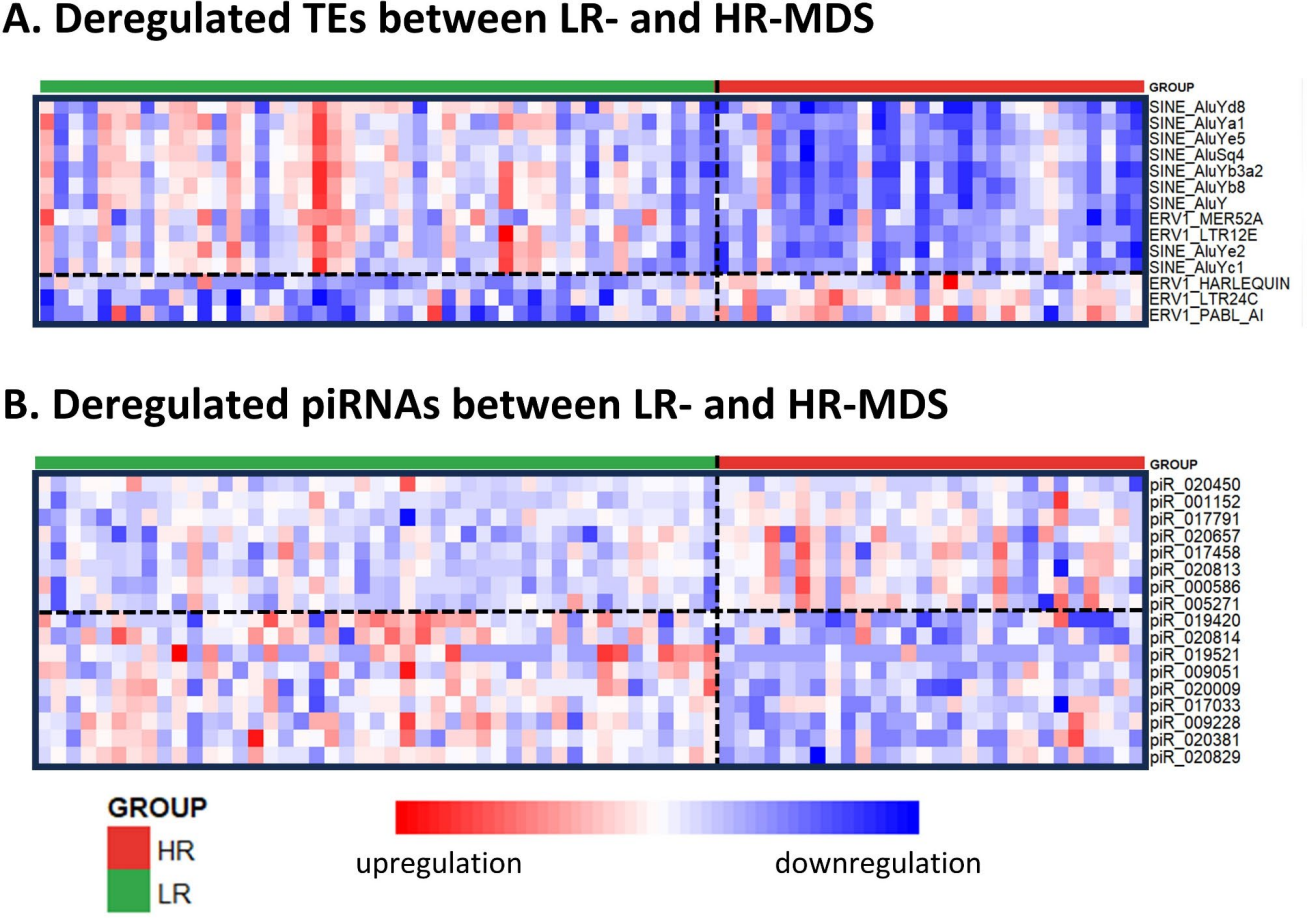
Dysregulated expression of TEs and piRNAs in LR-MDS and HR-MDS. (**A**) Heatmap of the 14 most significantly dysregulated TEs identified via DEA between the LR-MDS patients and HR-MDS patients (FDR < 0.001). (**B**) Heatmap of the 17 most significantly dysregulated piRNAs in DEA performed between LR-MDS and HR-MDS patients (FDR < 0.05). The red color in the heatmaps indicates upregulation, the blue color indicates downregulation, and the color intensity indicates the level of dysregulation. The columns in the heatmap represent individual samples; green, LR-MDS; red, HR-MDS

We further explored the correlation of TE expression with the risk of MDS progression and revealed strong differences between LR-MDS patients and HR-MDS patients. With the same stringency cutoff (FDR < 0.05), 106 TEs were significantly dysregulated (48 upregulated and 58 downregulated TEs in HR-MDS). When we restricted the FDR cutoff value to 0.001, we identified the 14 TEs most significantly associated with the risk of disease progression. Three TEs (all ERV1: HARLEQUIN, LTR24C, and PABL_AI) were upregulated in the HR-MDS samples, and 11 TEs (SINEs: AluY, AluYb8, AluYd8, AluYe2, AluSq4, AluYa1, AluYb3a2, AluYc1, and AluYe5; ERV1s: LTR12E and LTR1A2) were downregulated (Fig. 1A). SI Table 4 shows the list of the dysregulated TEs (FDR < 0.001), including their classes, clades, normalized counts, log_2_FC values, and FDRs.

### General characteristics of piRNA expression in MDS

In parallel with the analysis of TEs, we also assessed the expression of piRNAs in CD34+ BM cells from the same cohort. We performed small RNA-seq analysis and identified 257 differentially expressed piRNAs in total. The most highly expressed piRNAs in the whole dataset included piR_018780, piR_017458, and piR_001042. DEA of piRNA levels was performed for the MDS patients and controls; the differences in expression for most assessed piRNAs were nonsignificant; only three piRNAs were significantly dysregulated (FDR < 0.05). The expression of piR_016659 and piR_016658 was upregulated, and the expression of piR_019368 was downregulated in MDS patients (SI Fig. 3).

Furthermore, we assessed changes in piRNA expression during MDS progression and revealed more pronounced differences between LR-MDS patients and HR-MDS patients. We identified 17 differentially expressed piRNAs (7 upregulated piRNAs and 10 downregulated piRNAs in HR-MDS) (Fig. 1B). SI Table 5 shows the list of the dysregulated piRNAs, including their normalized counts, log_2_FC values, and FDRs.

### Total TE and piRNA levels and risk of disease progression

**Fig. 2:**
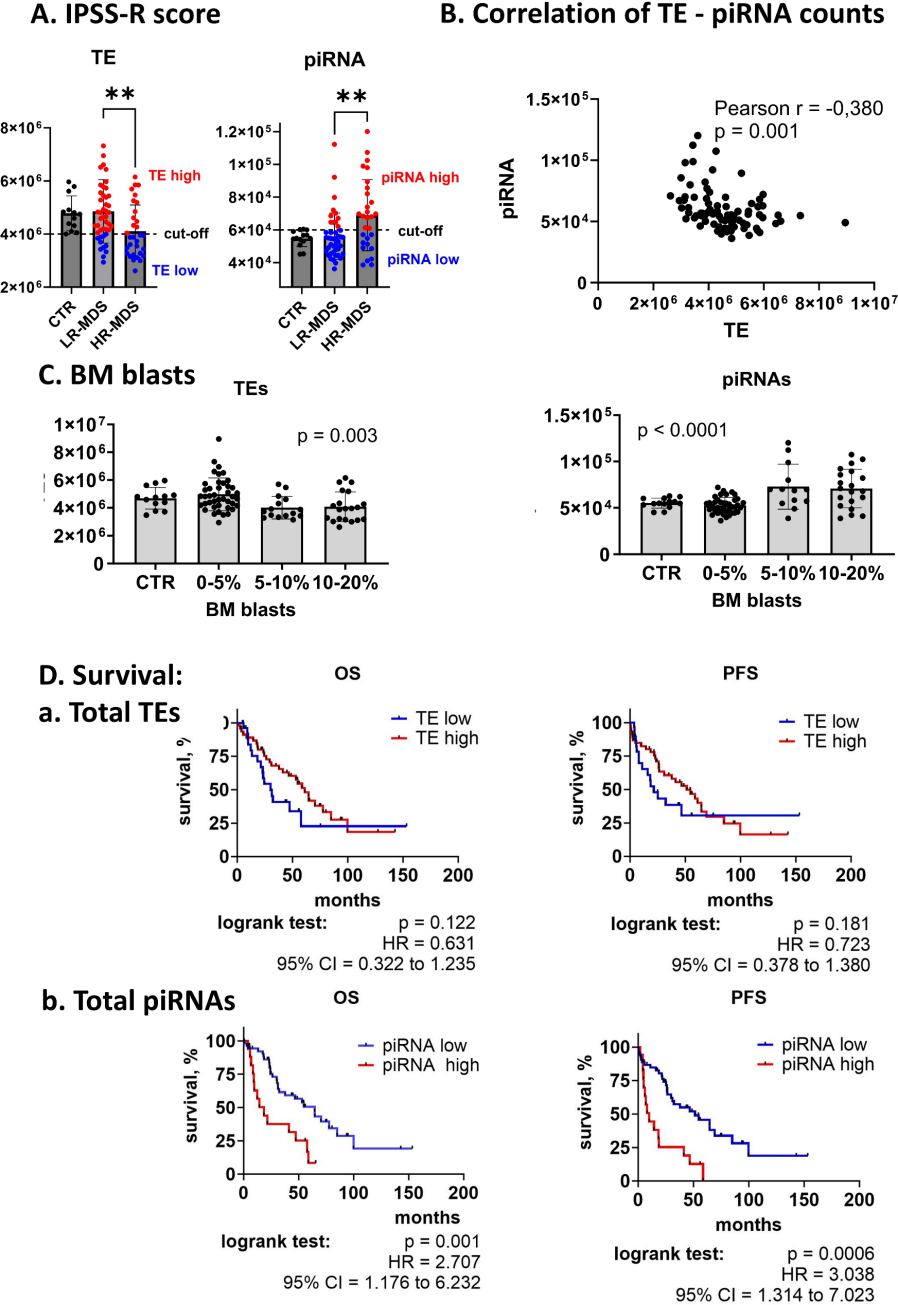
Overall TE and piRNA expression. Normalized levels of TEs and piRNAs from RNA-seq and small RNA-seq, respectively, are shown. (**A**) Differences in overall TE and piRNA levels between controls (CTR), LR-MDS patients, and HR-MDS patients as defined by the IPSS-R. Student´s t test was used to assess the statistical significance of the difference between the LR-MDS and HR-MDS groups. ** *p* < 0.01. (**B**) Correlation between overall TE and piRNA levels. Pearson’s test was performed to evaluate correlations. (**C**) Differences in total TE and piRNA levels in MDS patients stratified according to BM blast count. Welch´s ANOVA was used to assess the statistical significance between groups of samples. (**D**) Survival of MDS patients grouped according to the total levels of (**a**) TEs and (**b**) piRNAs. OS and PFS curves were plotted via the Kaplan‒Meier method. Patients were divided into separate “low” and “high” groups on the basis of TE or piRNA levels measured in CTR samples. Cutoff values were set as the minimum TE or maximum piRNA values detected in the CTR group as indicated in (**A**). Differences between the curves were compared via the log-rank test. HR – hazard ratio; CI – confidence interval

On the basis of the initial results showing some differences between LR-MDS and HR-MDS, we further focused on TE and piRNA levels with respect to the risk of disease progression in more detail. First, we assessed total TE and piRNA levels (i.e., total normalized TE and piRNA counts from RNA-seq and small RNA-seq data, respectively). The data revealed that the patients with HR-MDS presented significantly lower total TE expression than the patients with LR-MDS and controls did (*p* = 0.005). In contrast, a significant increase in total piRNA levels was found in the HR-MDS patients compared with those in the LR-MDS patients and controls (*p* = 0.003; Fig. 2A). Finally, we observed a moderate negative correlation between the two variables (Pearson *r* = -0.380, *p* = 0.001; Fig. 2B), indicating a known biological relationship between TEs and piRNA molecules.

### Total TE and piRNA levels and clinical parameters in MDS

Because MDS is a highly heterogeneous disease with many variable clinicopathological features, we further explored the relationships between TE and piRNA expression and the clinical and molecular parameters commonly associated with MDS diagnosis. Using the Pearson correlation test, we observed that the majority of clinicopathological features, including hemoglobin levels, neutrophil and platelet counts, sex, and age, did not correlate with either TE or piRNA levels. However, we demonstrated a significant correlation between the BM blast count and both TE levels (Pearson *r* = -0.330; *p* = 0.003) and piRNA levels (Pearson *r* = 0.354; *p* = 0.002); higher BM blast counts (> 5%) were significantly associated with reduced TE levels and increased piRNA expression (Fig. 2C).

With respect to the WHO classification, we observed a significant reduction in TE levels in patients with advanced subtypes of MDS, i.e., in MDS patients with excess blasts (EB1 and EB2) compared with those in the control group and patients with early subtypes. These results were consistent with the low TE levels observed in patients with intermediate, high and very high IPSS-R categories and in patients with higher blast counts (> 5% of BM blasts). In contrast, piRNA expression was increased in these patient groups (EB1 and EB2 diagnostic categories, intermediate to very high IPSS-R categories, and > 5% blasts) (SI Fig. 4).

Finally, we assessed whether the TE and piRNA profiles are related to a specific molecular MDS category (i.e., to some cytogenetic aberrations or somatic mutations recurrently found in MDS). However, sufficient patients for statistical comparison within our cohort were only available for two molecular features: (i) isolated del5q (13 patients with isolated del5q vs. 42 patients with a normal karyotype) and (ii) a somatic mutation of the *SF3B1* gene (10 patients with isolated mutations in the *SF3B1* gene vs. 16 patients with no mutation); the remaining patients had heterogeneous aberrations and/or mutational profiles. In the DEA, we identified 44 TEs and only three piRNAs that were dysregulated in MDS patients with del5q (compared with patients with a normal karyotype, SI Table 6). Only six TEs and three piRNAs were dysregulated in patients with mutated *SF3B1* (compared with patients with no mutation, SI Table 7), suggesting that there is no significant relationship between TE and piRNA expression and *SF3B1* mutation. However, further detailed analyses are necessary for a deeper understanding of the relationship between TE and piRNA expression and the genetic background of MDS.

### Total TE and piRNA levels and patient survival

Because both total TE and piRNA expression significantly changed in HR-MDS patients, we assessed whether their dysregulation was also associated with patient survival. On the basis of TE levels, we divided patients into two separate groups, “TE high” and “TE low”, and plotted Kaplan‒Meier curves for OS and PFS. The cutoff value was set to the minimum TE level detected in the CTR samples (see Fig. 2A). Although there was a slight trend toward adverse outcomes in patients with low TE levels, the differences in OS and PFS between “TE low” and “TE high” patients were not statistically significant (*p* = 0.122 and *p* = 0.181, respectively).

However, the same type of analysis of piRNA data revealed that high total piRNA levels are strongly associated with adverse outcomes (both OS and PFS) in MDS patients. The cutoff value used for this analysis was set to the maximum piRNA level detected in CTR samples (see Fig. 2A). The hazard ratio (HR) for the OS of patients with high piRNA levels was 2.707 (95% confidence interval, 95% CI = 1.176 to 6.232; *p* = 0.001). The median OS of patients with low piRNA expression reached 64.5 months, whereas that of patients with high piRNA expression was only 18.4 months. The HR for PFS reached 3.038 (95% CI = 1.3147.023, *p* = 0.0006), and the median PFS times for these two patient groups were 51.4 (low piRNA group) and 10.1 months (high piRNA group) (Fig. 2D).

### Individual TEs and piRNAs and patient outcomes

**Fig. 3:**
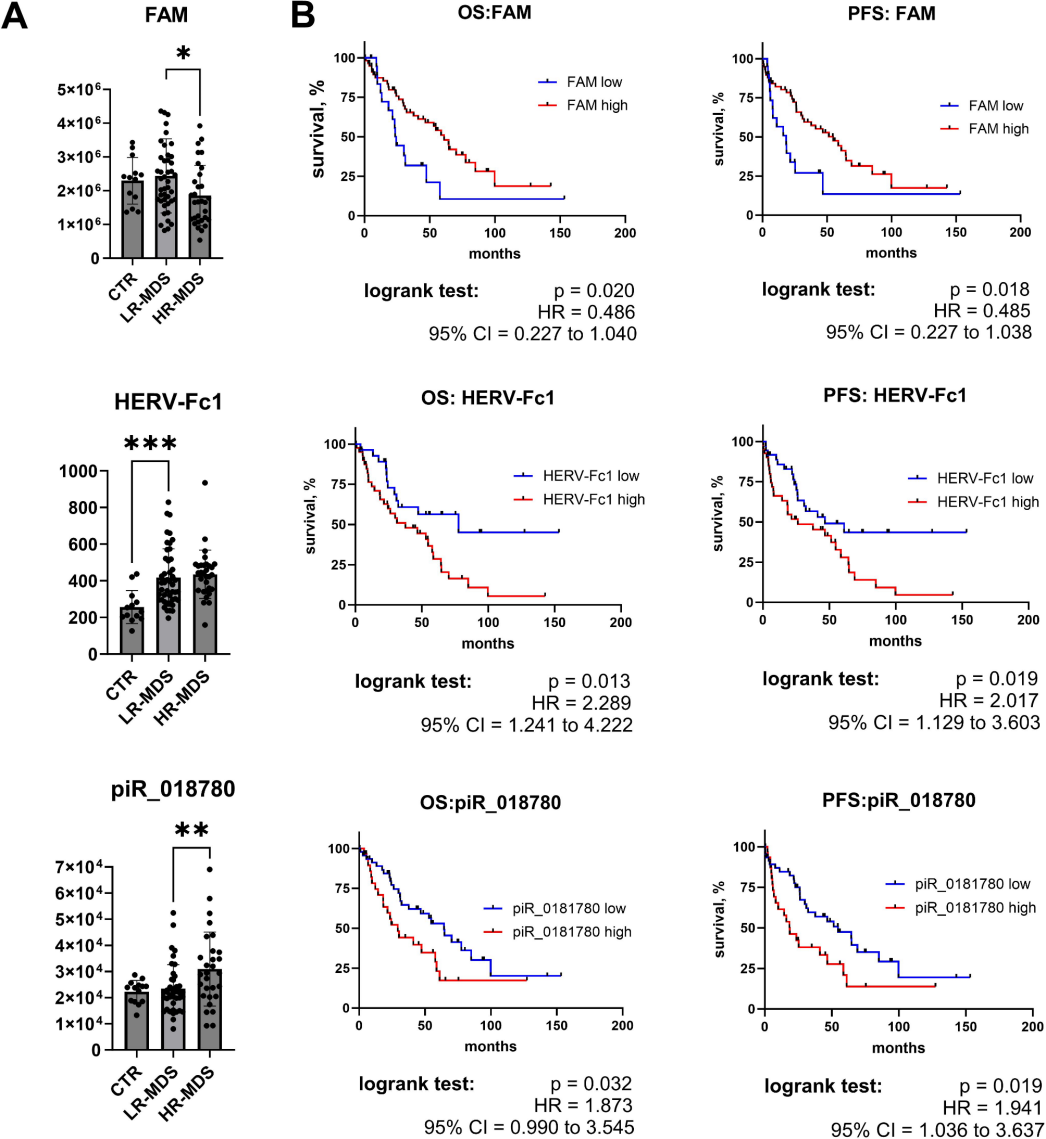
Expression of FAM, HERV-Fc1, and piR_018780. (**A**) Differences in the levels of FAM, HERV-Fc1, and piR_018780 in the control (CTR), LR-MDS, and HR-MDS groups as defined by IPSS-R (normalized levels in RNA-seq or small RNA-seq data). Student´s t test was used to assess the statistical significance of differences between two groups. *** FDR < 0.001; ** FDR < 0.01; * FDR < 0.05. (**B**) Survival of MDS patients grouped according to the levels of FAM, HERV-Fc1, and piR_018780. OS and PFS curves were plotted via the Kaplan‒Meier method. Patients were divided into separate “low” and “high” groups on the basis of the levels measured in CTR samples. The cutoff values were set as the minimum TE or maximum piRNA values detected in the CTR group. Differences between the curves were compared via the log-rank test. HR – hazard ratio. CI – confidence interval

To identify an individual molecular prediction marker related to TE/piRNA dysregulation, we further assessed the associations of the levels of individual TE and piRNA molecules with MDS progression and survival. For this purpose, we preselected FAM (a member of the SINE clade of non-LTR retrotransposons, one of the most highly expressed TEs, reduced in LR-MDS), HERV-Fc1 (a member of the ERV1 clade of LTR retrotransposons, the most significantly dysregulated TE, increased in MDS samples compared with controls), and piR_018780 (one of the most highly expressed piRNAs, increased in HR-MDS). Figure 3A shows changes in their levels in the control, LR-MDS, and HR-MDS groups.

Kaplan–Meier curves plotted for both OS and PFS revealed that the levels of each of the three molecules can significantly stratify MDS patients (Fig. 3B). Like those with increased total piRNA and reduced TE counts, patients with increased piR_018780 or reduced FAM levels had inferior survival (piR_018780 - OS: *p* = 0.031, PFS: *p* = 0.019; FAM - OS: *p* = 0.020, PFS: *p* = 0.018). However, lower HERV-Fc1 expression was associated with significantly better patient survival (OS: *p* = 0.013; PFS: *p* = 0.019).

To validate the NGS data, we reanalyzed the presence and expression of the two most highly expressed molecules, FAM and piR_018780, in a subset of samples (15 MDS patients and 5 controls) via RT‒qPCR. A high correlation of the results (FAM: Pearson *r* = 0.782, *p* = 0.0001; piR_018780: Pearson *r* = 0.702, *p* = 0.002) confirmed the accuracy of the NGS outputs and the chosen bioinformatic approaches (SI Fig. 5).

### *PIWIL* expression and patient outcome

**Fig. 4:**
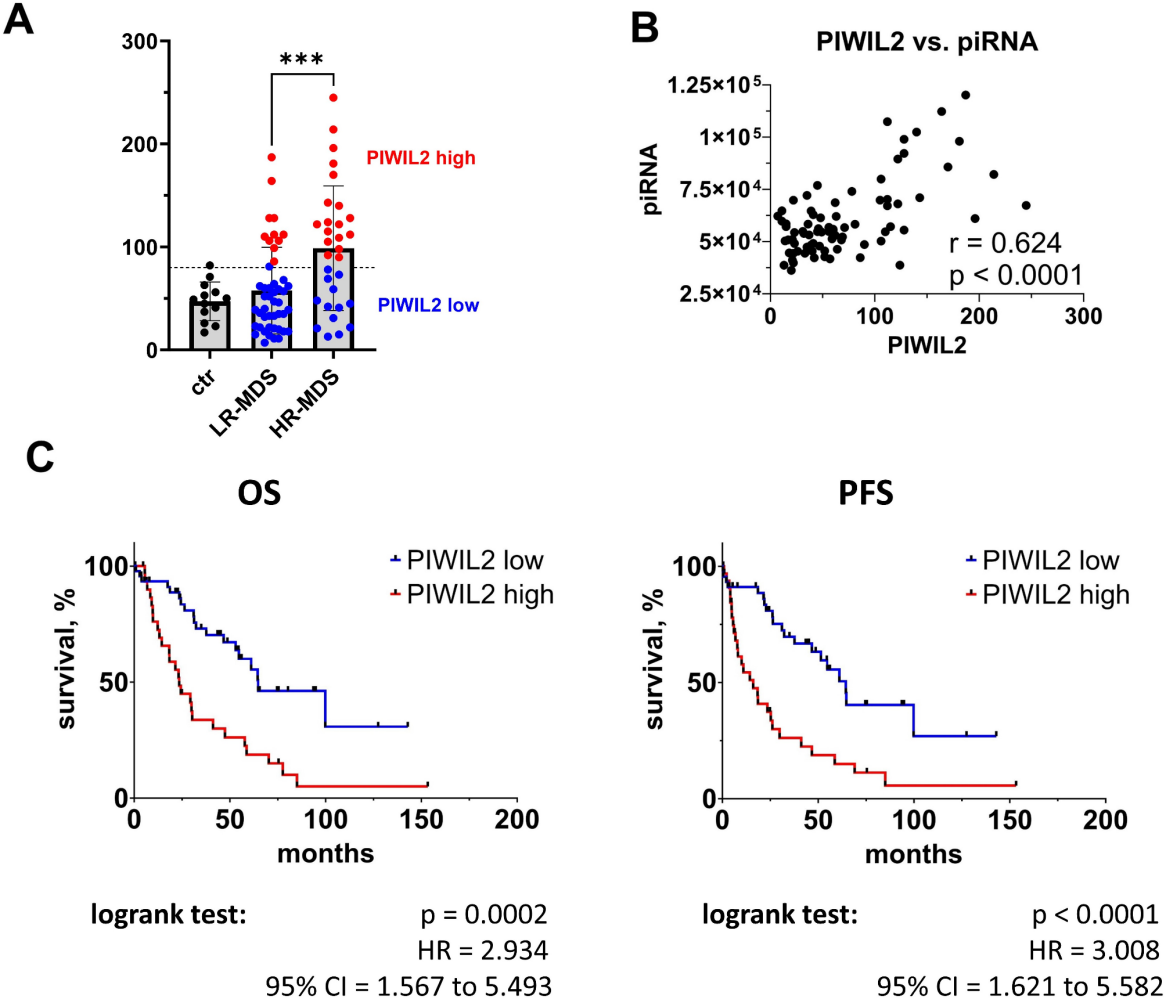
*PIWIL2* expression in MDS. (**A**) Differences in *PIWIL2* expression in the CTR, LR-MDS, and HR-MDS groups as defined by the IPSS-R. Normalized levels from the RNA-seq data are shown. Student´s t test was used to assess the statistical significance of differences between the LR-MDS and HR-MDS groups. *** *p* < 0.001. (**B**) Correlation between *PIWIL2* expression and overall piRNA levels. Pearson’s test was performed to evaluate the correlation. (**C**) Survival of MDS patients grouped according to the *PIWIL2* level. OS and PFS curves were plotted via the Kaplan‒Meier method. Patients were divided into separate “low” and “high” groups on the basis of *PIWIL2* levels measured in CTR samples. The cutoff values were set as the maximum values detected in the CTR group, as indicated in (**A**). Differences between the curves were compared via the log-rank test. HR – hazard ratio. CI – confidence interval

Four *PIWI* genes are present in humans (*PIWIL1-4*) [31]. Because the upregulation of *PIWIL*s is correlated with poor clinical outcomes in various cancers [32], we assessed their expression in MDS patients. According to RNA-seq, only *PIWIL2* and *PIWIL4* were detected in MDS CD34+ cells. Although the expression of these two genes did not significantly differ between the CTR and patient samples, we detected a twofold increase in the *PIWIL2* level in HR-MDS compared with that in LR-MDS (*p* < 0.001) (Fig. 4A). In contrast, the slight increase in the *PIWIL4* level observed in the HR-MDS was not significant (*p* = 0.0981) (SI Fig. 6A). Furthermore, we compared the expression levels of *PIWIL*s and piRNAs and observed a strong correlation between the *PIWIL2* level and total piRNA level (Pearson *r* = 0.624, *p* < 0.001; Fig. 4B), whereas the level of *PIWIL4* was independent of the total piRNA level (Pearson *r* = -0.073, *p* = 0.517; SI Fig. 6B). Because piRNA expression appeared to be a significant predictor of MDS patient survival according to our data, we plotted Kaplan–Meier curves for patients divided on the basis of the expression level of the two *PIWIL*s. Like in the preceding univariate analyses, cutoffs for separating patients into “high *PIWIL*” and “low *PIWIL”* groups were set to the maximum level detected in the CTR group. A log-rank test revealed that a high *PIWIL2* level (Fig. 4C), but not a high *PIWIL4* level (SI Fig. 6C), was significantly associated with both OS and PFS. The median OS of MDS patients with high *PIWIL2* levels was 23.5 months, whereas the OS of patients with low *PIWIL2* was 64.7 months (HR = 2.934, 95% CI = 1.5675.493, p = 0.0002). The median PFS times were 16.1 months (high *PIWIL2*) and 64.5 months (low *PIWIL2)* (HR = 3.008, 95% CI = 1.621 to 5.582, p < 0.0001), suggesting that *PIWIL2* expression might be a promising molecular marker of MDS prognosis.

The association of *PIWIL2* with patient outcome was further validated in an independent cohort of 72 MDS patients and 11 controls, which was previously established by Szikszai et al. [25]. Here, we measured the expression of *PIWIL2* in CD34+ BM cells from these samples via ddPCR. Univariate analyses of the output data confirmed that high *PIWIL2* expression is a strong predictor of short OS (median OS: high *PIWIL2* = 12.2 months and low *PIWIL2* = 50.9 months, HR = 3.379, 95% CI = 1.364 to 8.372, *p* < 0.0001) and short PFS (median PFS: high *PIWIL2* = 9.9 months and low *PIWIL2* = 43.1 months HR = 3.014, 95% CI = 1.264 to 7.190, *p* = 0.0002) (SI Fig. 7).

### Multivariate analysis revealed that *PIWIL2* expression is a prognostic marker in MDS

Finally, to determine whether any of the molecular variables evaluated might serve as prognostic markers for MDS outcome, we performed Cox multivariate analysis (MVA). For the analysis, we combined variables commonly used for IPSS-R risk stratification (e.g., blast count, hemoglobin level, neutrophil and platelet counts, and cytogenetics) with age, sex, and the expression values of selected individual molecules that showed predictive power in the univariate analyses described above (piR_018780, FAM, HERV-Fc1, and *PIWIL2*). We did not include total TE and piRNA levels because their evaluation requires nonstandard laboratory-intensive quantification and thus cannot be tested in routine clinical measurements.

The prognostic value of the abovementioned parameters was first analyzed separately via a series of univariate analyses. The most significant variables were then included in a single MVA, and the stepwise selection method was applied to retain only the independent variables significantly contributing to the predictive power of the resulting model. MVA revealed that the predominant variables predictive of inferior OS in MDS patients were high *PIWIL2* levels, low platelet counts, and increased age. For PFS, female sex was added to these predictors; it was a favorable predictor that reduced the HR. The HRs, confidence intervals, and *p* values of the variables included in the final predictive models are summarized in Table 1. Among the four molecules we tested, high *PIWIL2* levels were the only potential marker retained in the final predictive models for both OS (HR = 3.197, 95% CI = 1.618–6.314, *p* = 0.0008) and PFS (HR = 3.637, 95% CI = 1.771–7.470, *p* = 0.0004).

The resulting set of parameters predictive of MDS outcome is particularly interesting considering that the *PIWIL2* level and blast count were correlated (Pearson *r* = 0.284, *p* = 0.011) (SI Fig. 8) and thus likely dependent on one another. Therefore, the inclusion of *PIWIL2* and the exclusion of the blast count from the final set of parameters highlights the strong predictive power of *PIWIL2*.

### Biological processes related to TE and piRNA dysregulation

**Fig. 5:**
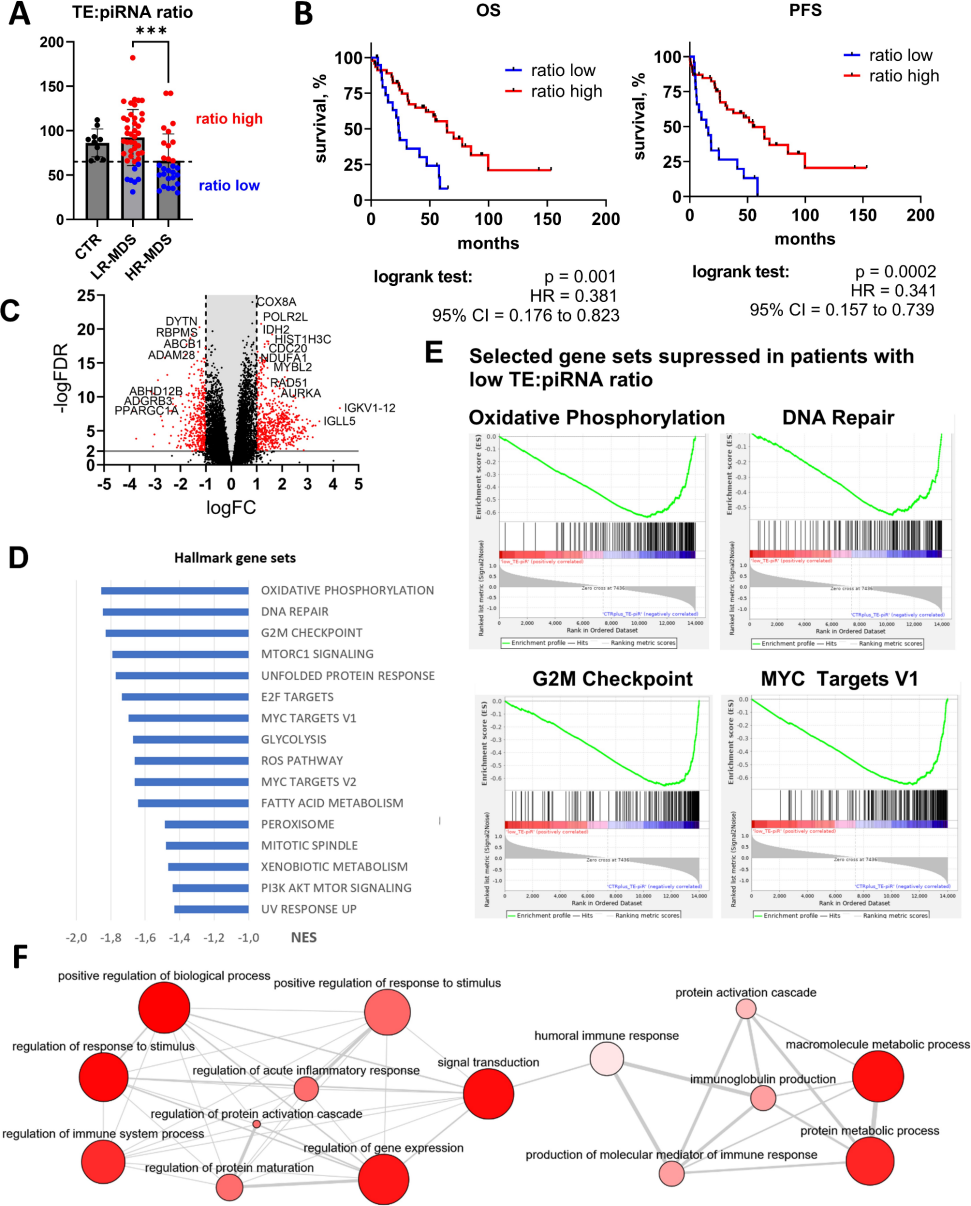
Biological processes related to TE and piRNA dysregulation. (**A**) TE: piRNA ratio and its values in the CTR, LR-MDS, and HR-MDS groups as defined by the IPSS-R. The ratio was calculated from total normalized TE and piRNA levels in the RNA-seq and small RNA-seq data. Student´s t test was used to assess the statistical significance of differences between the LR-MDS and HR-MDS groups. *** *p* < 0.001. (**B**) Survival of MDS patients grouped according to the TE: piRNA ratio. OS and PFS curves were plotted via the Kaplan‒Meier method. Patients were divided into “low-ratio” (blue) and “high-ratio” (red) groups on the basis of the minimum value of the ratio observed in controls, as indicated in (**A**). Differences between the curves were compared via the log-rank test. HR – hazard ratio. CI – confidence interval. (**C**) Volcano plot of differentially expressed protein-coding genes between low- and high-TE: piRNA ratio samples. The red points indicate the most significantly dysregulated genes (FDR < 0.001 and |logFC| > 1). The most upregulated genes in samples with a high TE: piRNA ratio (i.e., downregulated in samples with a low ratio) are located toward the right and vice versa. X-axis: binary logarithm of the fold change; y-axis: negative logarithm of the FDR value. (**D**) Significantly enriched (FDR < 0.2) hallmark gene sets identified via GSEA of genes that were differentially expressed between low- and high-ratio samples. NES – normalized enrichment score. (**E**) Four selected gene sets significantly enriched in samples with a high TE: piRNA ratio (i.e., suppressed in samples with a low ratio) in the GSEA output. (**F**) Pathway analysis of GO biological processes by the GOrilla enrichment tool on a subset of significantly (FDR < 0.001 and |logFC| > 1) downregulated genes in samples with a low TE: piRNA ratio. Visualization and reduction of redundant GO terms were performed via the Revigo tool. The color intensity of the bubble corresponds to the significance of the *p* value and its size to the LogSize value for a given GO term

Here, we showed that low TEs and particularly high piRNA levels are associated with the risk of progression and adverse outcomes in MDS patients. Therefore, functional changes related to this dysregulation are important. Because piRNA and TE levels are correlated and their dysregulation specifically co-occurs in a substantial number of HR-MDS patients, we introduced a TE: piRNA ratio, which helped us define samples whose variables were altered simultaneously. On the basis of the TE: piRNA ratios observed in the CTR samples, we split our MDS patient cohort into (i) a group of patients whose ratios were comparable to those of the CTR samples (“high ratio”; 34 LR- and 11 HR-MDS patients) and (ii) a group of patients whose ratios were below the CTR minimum (“low ratio”; 8 LR- and 17 HR-MDS patients) (Fig. 5A). Kaplan–Meier analysis together with the log-rank test revealed that patients with a low TE: piRNA ratio (i.e., low TE and high piRNA expression) had significantly worse OS and PFS (*p* = 0.0014 and *p* = 0.0002, respectively; Fig. 5B).

DEA of PCGs revealed a substantial difference in gene expression profiles between low and high TE: piRNA ratio samples (1,011 dysregulated genes with FDR < 0.05 and |log_2_FC| > 1, Fig. 5C). Subsequent pathway analysis via GSEA of hallmark gene sets revealed strong suppression of multiple cellular processes in samples with low ratios. These processes included oxidative phosphorylation (OXPHOS), glycolysis, DNA repair, the reactive oxygen species (ROS) pathway, the G2M cell cycle checkpoint, mitosis, PI3K/AKT/mTORC signaling, and targeting of the MYC transcription factor (Fig. 5D and E). The most significantly downregulated genes involved in these processes included *COX8A*,* COX6A1*,* IDH2*, multiple *NDUF* genes, *GPX4*,* RAD51*,* CDC20*,* CCNA2*,* CDK1/2/4*,* PCNA*,* AURKA*,* E2F*, multiple *HIST* genes, *MYBL2*,* AKT1*, and *MYD88*. While many key cellular processes were suppressed, no process was significantly activated despite the multitude of significantly upregulated genes (e.g., *ABCB1*,* ABHD12B*,* ADAM28*,* ADGRB3*,* DYTN*,* PPARGS1A*, and *RBPMS*). To assess the validity of our conclusions, we also performed GSEA based separately on piRNA or TE levels, and these experiments mirrored the results obtained for the TE: piRNA ratio, confirming the appropriateness of the ratio introduction (data not shown).

We also explored GO biological process terms associated with TE: piRNA dysregulation via the GOrilla enrichment analysis tool followed by GO reduction and visualization via Revigo. Analysis of the most significantly differentially expressed genes (FDR < 0.001 and |log_2_FC| > 1) revealed strong suppression of processes connected with the immune response, such as the regulation of immune system processes, immunoglobulin production, inflammation, and complement activation (Fig. 5F). This dysregulation was driven by the marked suppression of many immunoglobulin genes (the three most suppressed IG genes were *IGKV1-12*,* IGLL5*, or *IGLV2-23*). Interestingly, a significant reduction in the expression of the recombination-activating genes *RAG1* and *RAG2* was also observed in this analysis.

### Gene networks for piR_018780, FAM, HERV-Fc1, and *PIWIL2*

**Fig. 6:**
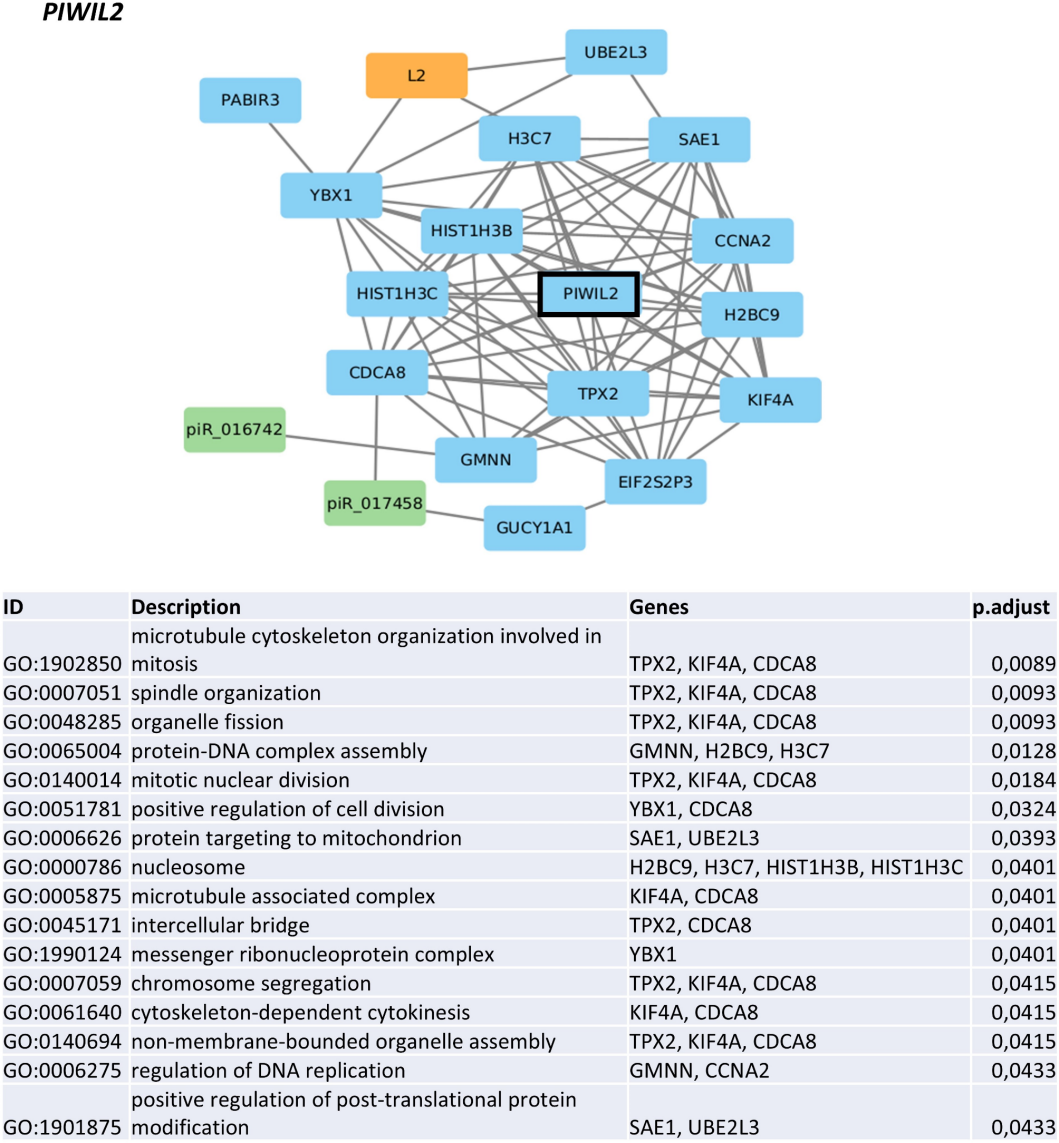
Gene network graph and pathway enrichment data generated for the *PIWIL2* gene. The graph composed of PCGs, TEs, and piRNAs was computationally modeled via RNA-seq and small RNA-seq data on the basis of pairwise correlations. The enrichment analysis was based on annotations of PCGs included in the graph, and significant GO terms are listed in the corresponding table (adjusted *p* < 0.05)

Finally, to elucidate the possible functions of the four individual molecules studied above (*PIWIL2*, piR_018780, FAM, and HERV-Fc1) in MDS progression, we computationally modeled their gene expression networks. Enrichment analyses, which were subsequently performed on the basis of annotations of PCGs included in each network, indicated possible relationships between these molecules and specific cellular processes. Network graphs composed of PCGs, TEs, and piRNAs showing the genes and pathways most significantly associated (adjusted *p* < 0.05) with each of these four molecules are included in Fig. 6 (graph for *PIWIL2*) and SI Fig. 9A-C (graphs for piR_018780, FAM, and HERV-Fc1).

Network modeling yielded similar results for *PIWIL2* and piR_018780. PCGs correlated with both of these factors were predominantly associated with GO terms related to cell division, such as chromosome segregation, spindles, or microtubule polymerization. The cell division-related network neighbors of *PIWIL2* were *TPX2*,* KIF4A*, and *CDCA8*. In addition, histones involved in nucleosome organization (*H2BC9*,* H3C7*,* HIST1H3B*, and *HIST1H3C*) and the regulation of multiple steps of the gene expression process *(YBX1*,* GMNN*,* CCNA2*,* SAE1*, and *UBE2L3*) were related to *PIWIL2* (Fig. 6). The interconnection between *PIWIL2* and TE/piRNA dysregulation is further supported by an overlap of the *PIWIL2*-networked genes with those found among the most downregulated genes in patients with low TE: piRNA ratios (*HIST1H3B*,* HIST1H3C*, and *CCNA2*).

With respect to piR_018780, its PCG network neighbors related to cell division processes were *CDC20*,* HAUS8*,* TUBG1*, and *KIF2C.* The other genes most coexpressed with piR_018780 encoded several RNA-binding proteins related to mRNA splicing or the telomerase complex (*YBX1*,* NOP10*, and *SART3*) (SI Fig. 9A).

Genes involved in networks constructed for the TEs (FAM and HERV-Fc1) were not enriched in many associated GO terms. FAM expression was correlated with the expression of several genes related to transcription (*YBX1*,* ELOB*, and *NELFB*) (SI Fig. 9B). The *TBC1D7* gene, which functions in mTORC signaling (SI Fig. 9C), was among the network neighbors of HERV-Fc1.

## Discussion

Our current understanding of HSC differentiation and abnormalities in this process that lead to MDS is mainly based on knowledge of PCGs. The possible impacts of TE mobilization and piRNA expression on MDS pathogenesis have not been a key research focus thus far. To further elucidate the possible roles of TEs and piRNAs in MDS and leukemogenesis, we analyzed the expression of TEs and piRNAs in MDS patients at different stages of the disease.

TE mobilization is generally elevated in advanced cancers, contributing to disease progression [6]. However, our data suggest an opposite trend with TE suppression in HR-MDS compared with LR-MDS. This observation is in agreement with an earlier study by Colombo et al. [7], who described TE suppression along with disease progression and proposed TE activity as a potential mechanism for immune-mediated elimination of leukemic cells in LR-MDS. Thus, the causes and consequences of this TE dysregulation in MDS were the main focus of our study.

Among the major regulators of TE expression are piRNAs [8]. These molecules inhibit TE mobilization and their dysregulation may represent an important regulatory layer affecting TE activity in MDS. Our parallel exploration of TE and piRNA levels revealed the opposite trend of their expression, with increased piRNA expression in HR-MDS patients with reduced TE levels, which is consistent with the inhibitory role of piRNAs in TE regulation. Moreover, high piRNA expression was strongly associated with worse outcomes in MDS patients. Our results indicate that the piRNA level seems to be a good predictor of patient progression and survival; however, it is not easily applicable in clinical practice, as measuring the total piRNA level via NGS is expensive and laborious. Therefore, we searched for an individual molecule that is easily quantified via a standard method of molecular genetics that would reflect the total piRNA level in its predictive power and substitute it in the modeling of risk prediction. Among the molecules we chose for testing, *PIWIL2* showed the most significant results. Its expression proved to be one of the most significant independent variables in multivariate analysis, performing better than the majority of clinical variables, even blast counts. Another advantage of *PIWIL2* is that it is a common PCG, so it is a more appropriate predictive marker than piRNAs, which typically exhibit only slight differences in length and sequence; for example, many piRNAs differ from others only by a single nucleotide [23].

*PIWIL2* dysregulation has been shown to influence several fundamental cellular processes, including proliferation, cell cycle progression, and the DNA damage response, across multiple cancer types [33]. For example, *PIWIL2* upregulation promotes glioblastoma cell proliferation and migration, and its level has been correlated with poor prognosis [34]. In vitro knockdown of *PIWIL2* in glioma cells was shown to induce cell cycle arrest and increase apoptosis, and *PIWIL2* silencing suppressed the migration of glioma cells [34]. Similarly, knockdown of *PIWIL2* in cervical cancer cells led to a marked reduction in proliferation and colony formation, in vivo tumorigenicity, chemoresistance, and the proportion of cancer stem-like cells [35]. *PIWIL2* overexpression in cervical cancer initiates metabolic reprogramming via *PDK1* upregulation, causing a shift in cellular metabolism from oxidative phosphorylation to glycolysis and the sequential activation of PI3K/AKT/mTOR signaling [36]. Similarly, the overexpression of *PIWIL2* promoted cell proliferation, and *PIWIL2* interference arrested non-small cell lung cancer cells at the G2/M stage [37]. Moreover, the overexpression of *PIWIL2* in some cancers leads to cellular cisplatin resistance, possibly because increased chromatin condensation affects normal DNA repair [38]. Taken together, these findings align with our pathway analysis results, which suggest that *PIWIL2* may also play a role in regulating cell proliferation in HR-MDS.

The role of *PIWIL2* in MDS pathogenesis may be further underscored by its coexpression with the *YBX1* gene. This gene was present in three of our coexpression network modules (those of *PIWIL2*, piR_018780, and FAM). The gene encodes a DNA- and RNA-binding protein implicated in numerous cellular processes, including the regulation of gene expression, pre-mRNA splicing, and DNA repair. Aberrant expression of this gene is associated with proliferation in various cancers [39]. *YBX1* has also been demonstrated to be specifically required for maintaining myeloid leukemia cell survival [40]. Collectively, while direct functional experiments in MDS are currently lacking, our data, combined with insights from other cancers, strongly support the hypothesis that *PIWIL2* upregulation contributes to MDS pathogenesis through multiple interconnected pathways and that its upregulation in HR-MDS could serve not only as a prognostic predictor but also as a novel approach for personalized therapy of MDS in the future.

The major function of piRNAs is the suppression of TE activity; however, both piRNAs and TEs have been shown to directly regulate the expression of many PCGs [41, 42]. In this context, these molecules may directly alter the transcriptional network, which may further affect the differentiation of HSCs and leukemogenesis. We investigated the cellular processes associated with TE and piRNA dysregulation in MDS and revealed that patients with dysregulated TE and piRNA levels (irrespective of their disease subtype or IPSS-R risk score) showed strong suppression of multiple genes involved in immune response processes. The suppression of the immune response we observed in patients with low TEs and high piRNA levels supports the abovementioned hypothesis that while active TEs observed in LR-MDS trigger immune-mediated clearance of cancer cells, TE suppression in HR-MDS may enable cells to escape the immune system [7, 43].

Among the dysregulated immune-related processes, strong suppression of immunoglobulin production was particularly apparent in patients with low TE levels and high piRNA levels. Although immunoglobulins are produced primarily by B lymphocytes and plasma cells, they can also be expressed in myeloblasts and have unique V(D)J rearrangements [44]. AML-derived immunoglobulins are involved in the proliferation, apoptosis, and migration of leukemic cells, and their high levels may play a role in AML pathogenesis and progression, potentially serving as a marker for monitoring residual disease [44]. However, here, we demonstrated the opposite observation: immunoglobulin production was suppressed with MDS progression. Interestingly, we also observed a significant reduction in the recombination-activating genes *RAG1* and *RAG2*, which are responsible for V(D)J recombination of immunoglobulin genes and are evolutionarily related to transposons [45].

TE and piRNA dysregulation is also accompanied by the suppression of mitochondrial energy metabolism, DNA repair, and cell cycle genes. In this context, it has recently been demonstrated that progenitor cells of LR-MDS patients who undergo accelerated progression exhibit transcriptome patterns of a quiescent-like cell state, which is characterized by reduced HSC differentiation and suppression of energy metabolism and the DNA damage response. In contrast, cells from patients with stable LR-MDS (without rapid progression) presented transcriptional signatures of actively cycling progenitors with upregulation of cell cycle checkpoints and regulation of immune system processes, presumably inducing a tumor-suppressing response [46]. The similarities in the dysregulated pathways between these two studies support our conclusions that suppressed TE and increased piRNA activities are linked with the MDS phenotype of rapid progression and that this phenotype is enabled by disruption of the antitumor response.

Finally, the therapeutic potential of targeting dysregulated molecules within the TE‒piRNA axis in MDS should also be considered. Notably, *PIWIL2* has been proposed as a promising target in different types of cancer because of its capacity to promote cell proliferation and inhibit apoptosis [34, 47]. In colorectal cancer, Farahani et al. reported that the *PIWIL2* gene is overexpressed in cancerous tissues but absent in normal tissues, offering a selective advantage for targeted therapy [47]. However, not only proteins but also various classes of noncoding RNAs, particularly long noncoding RNAs, circular RNAs, and miRNAs, have been suggested as molecular targets, rapidly expanding the scope of anticancer therapy in recent years [48]. Moreover, increasing evidence highlights the role of noncoding RNA crosstalk and its dysregulation in driving chemotherapy resistance or sensitizing resistant cells, garnering substantial interest within the cancer research community [49]. For example, recent findings have shown that interactions of the circular RNA circVDAC3 may help overcome the resistance to trastuzumab deruxtecan in breast cancer therapy [50]. Additionally, piRNAs are gaining attention as potential targets for anticancer therapy [51, 52]. However, their practical applications remain limited due to a lack of understanding of their regulatory mechanisms and challenges in achieving therapeutic specificity; for example, sequence similarity between target piRNAs and other RNA molecules poses a major hurdle, risking off-target effects and unintended modulation of related pathways.

## Conclusions

Our current study provides new knowledge about the involvement of TEs and piRNAs in MDS and shows that their dysregulation is associated with disease progression in HR-MDS. Comprehensive parallel analyses of TE and piRNA expression profiles in CD34+ HSCs from MDS patients offer original insights into the interplay between TEs and piRNAs in this disease, with findings revealing a significant inverse correlation between TE and piRNA expression in HR-MDS. Importantly, the identification of *PIWIL2* upregulation as a potential independent prognostic marker in MDS highlights a novel molecular pathway with strong implications for both prognostic and therapeutic strategies. To conclude, this study provides an indispensable resource for future research that may help to identify the causes and consequences of TE and piRNA dysregulation, define their precise role in disease progression, and elucidate their potential for future therapeutic implications.

## Electronic supplementary material

Below is the link to the electronic supplementary material.

Supplementary Material 1

## Data Availability

Raw NGS data from RNA-seq and small RNA-seq were deposited in the NCBI SRA database (BioProject ID PRJNA1172695).

## Abbreviations

AML: Acute myeloid leukemia
BM: Bone marrow
CD34+: Cluster of differentiation 34 positive
CI: Confidence interval
CTR: Control
ddPCR: Droplet digital PCR
DEA: Differential expression analysis
EB: Excess blasts
ERV: Endogenous retrovirus
FAM: Fossil Alu monomer
FC: Fold change
FDR: False discovery rate
GO: Gene Ontology
GSEA: Gene set enrichment analysis
Herv-Fc1: Human endogenous retrovirus Fc1
HR: Hazard ratio
HR-MDS: Higher-risk myelodysplastic neoplasms
HSC: Hematopoietic stem cell
IPSS-R: Revised International Prognostic Scoring System
LINE1: Long interspersed nuclear element
log_2_FC: Binary logarithm of the fold change
LR-MDS: Lower-risk myelodysplastic neoplasms
MDS: Myelodysplastic neoplasms
miRNA: MicroRNA
MNC: Mononuclear cell
MVA: Multivariate analysis
NGS: Next-generation sequencing
OS: Overall survival
OXPHOS: Oxidative phosphorylation
PCG: Protein-coding gene
PFS: Progression-free survival
piRNA: PIWI-interacting RNA
PIWIL: P-element-induced wimpy testis-like protein
RA: Refractory anemia
ROS: Reactive oxygen species
RT‒qPCR: Reverse transcription quantitative polymerase chain reaction
SINE: Short interspersed nuclear element
TE: Transposable element
UMI: Unique molecular identifier
WHO: World Health Organization

## References

[1] Khoury JD, Solary E, Abla O, Akkari Y, Alaggio R, Apperley JF, et al. The 5th edition of the World Health Organization Classification of Haematolymphoid Tumours: myeloid and Histiocytic/Dendritic neoplasms. Leukemia. 2022;36(7):1703–19. 35732831 10.1038/s41375-022-01613-1 PMC9252913

[2] Garcia-Manero G. Myelodysplastic syndromes: 2023 update on diagnosis, risk-stratification, and management. Am J Hematol. 2023;98(8):1307–25. 37288607 10.1002/ajh.26984 PMC12002404

[3] Gadji M, Pozzo AR. From cellular morphology to molecular and epigenetic anomalies of myelodysplastic syndromes. Genes Chromosomes Cancer. 2019;58(7):474–83. 30303583 10.1002/gcc.22689

[4] Payer LM, Burns KH. Transposable elements in human genetic disease. Nat Rev Genet. 2019;20(12):760–72. 31515540 10.1038/s41576-019-0165-8

[5] Tubbs A, Nussenzweig A. Endogenous DNA damage as a source of genomic instability in Cancer. Cell. 2017;168(4):644–56. 28187286 10.1016/j.cell.2017.01.002 PMC6591730

[6] Grundy EE, Diab N, Chiappinelli KB. Transposable element regulation and expression in cancer. FEBS J. 2022;289(5):11660–79. 10.1111/febs.15722 PMC11577309 33471418

[7] Colombo AR, Zubair A, Thiagarajan D, Nuzhdin S, Triche TJ, Ramsingh G. Suppression of transposable elements in leukemic stem cells. Sci Rep. 2017;7(1):7029. 28765607 10.1038/s41598-017-07356-9 PMC5539300

[8] Ozata DM, Gainetdinov I, Zoch A, O’Carroll D, Zamore PD. PIWI-interacting RNAs: small RNAs with big functions. Nat Rev Genet. 2019;20(2):89–108. 30446728 10.1038/s41576-018-0073-3

[9] Du WW, Yang W, Xuan J, Gupta S, Krylov SN, Ma X, et al. Reciprocal regulation of miRNAs and piRNAs in embryonic development. Cell Death Differ. 2016;23(9):1458–70. 26990662 10.1038/cdd.2016.27 PMC5072423

[10] Shaker FH, Sanad EF, Elghazaly H, Hsia SM, Hamdy NM. piR-823 tale as emerging cancer-hallmark molecular marker in different cancer types: a step-toward ncRNA-precision. Naunyn Schmiedebergs Arch Pharmacol. 2024;1–22. 10.1007/s00210-024-03308-z PMC11787197 39102033

[11] Ng KW, Anderson C, Marshall EA, Minatel BC, Enfield KSS, Saprunoff HL et al. Piwi-interacting RNAs in cancer: emerging functions and clinical utility. Mol Cancer 2016;15;5. 10.1186/s12943-016-0491-9 PMC4714483 26768585

[12] Ross RJ, Weiner MM, Lin H. PIWI proteins and PIWI-interacting RNAs in the soma. Nature. 2014;505(7483):353–9. 24429634 10.1038/nature12987 PMC4265809

[13] Cheng Y, Wang Q, Jiang W, Bian Y, Zhou Y, Gou A, et al. Emerging roles of piRNAs in cancer: challenges and prospects. Aging. 2019;11(21):9932–46. 31727866 10.18632/aging.102417 PMC6874451

[14] Jang HS, Shah NM, Du AY, Dailey ZZ, Pehrsson EC, Godoy PM, et al. Transposable elements drive widespread expression of oncogenes in human cancers. Nat Genet. 2019;51(4):611–7. 30926969 10.1038/s41588-019-0373-3 PMC6443099

[15] Colombo AR, Triche T, Ramsingh G. Transposable element expression in Acute myeloid leukemia transcriptome and prognosis. Sci Rep. 2018;8(1):1–10. 30401833 10.1038/s41598-018-34189-x PMC6219593

[16] Hrustincova A, Krejcik Z, Kundrat D, Szikszai K, Belickova M, Pecherkova P, et al. Circulating small noncoding RNAs have specific expression patterns in plasma and extracellular vesicles in myelodysplastic syndromes and are predictive of patient outcome. Cells. 2020;9(4):794. 32224889 10.3390/cells9040794 PMC7226126

[17] Beck D, Ayers S, Wen J, Brandl MB, Pham TD, Webb P et al. Integrative analysis of next generation sequencing for small non-coding RNAs and transcriptional regulation in myelodysplastic syndromes. BMC Med Genomics. 2011;4;19. 10.1186/1755-8794-4-19 PMC3060843 21342535

[18] Arber DA, Orazi A, Hasserjian R, Thiele J, Borowitz MJ, Le Beau MM, et al. The 2016 revision to the World Health Organization classification of myeloid neoplasms and acute leukemia. Blood. 2016;127(20):2391–405. 27069254 10.1182/blood-2016-03-643544

[19] Greenberg PL, Tuechler H, Schanz J, Sanz G, Garcia-Manero G, Solé F, et al. Revised international prognostic scoring system for myelodysplastic syndromes. Blood. 2012;120(12):2454–65. 22740453 10.1182/blood-2012-03-420489 PMC4425443

[20] Montoro MJ, Ortega M, Villacampa G, Bernal T, Pomares H, Mora Casterá E, et al. A revised International Prognostic Scoring System of 3.5 points stratifies patients with myelodysplastic syndromes into 2 risk categories. Blood. 2020;136(1):9–10.

[21] 21 Jeong HH, Yalamanchili HK, Guo C, Shulman JM, Liu Z. An ultra-fast and scalable quantification pipeline for transposable elements from next generation sequencing data. Pac Symp Biocomput. 2018;23:168–79. 29218879

[22] Wang YE, Kutnetsov L, Partensky A, Farid J, Quackenbush J. WebMeV: a cloud platform for analyzing and visualizing cancer genomic data. Cancer Res. 2017;77(21):11–4. 10.1158/0008-5472.CAN-17-0802 PMC5679251 29092929

[23] Sai lakshmi S, Agrawal S, piRNABank:. A web resource on classified and clustered Piwi-interacting RNAs. Nucleic Acids Res. 2008;36(1):173–7. 10.1093/nar/gkm696 PMC2238943 17881367

[24] Smith T, Heger A, Sudbery I. UMI-tools: modeling sequencing errors in Unique Molecular identifiers to improve quantification accuracy. Genome Res. 2017;27(3):491–9. 28100584 10.1101/gr.209601.116 PMC5340976

[25] Szikszai K, Krejcik Z, Klema J, Loudova N, Hrustincova A, Belickova M, et al. LncRNA profiling reveals that the deregulation of H19, WT1-AS, TCL6, and LEF1-AS1 is associated with higher-risk myelodysplastic syndrome. Cancers (Basel). 2020;12(10):1–21. 10.3390/cancers12102726 PMC7598221 32977510

[26] Subramanian A, Tamayo P, Mootha VK, Mukherjee S, Ebert BL, Gillette MA, et al. Gene set enrichment analysis: a knowledge-based approach for interpreting genome-wide expression profiles. Proc Natl Acad Sci U S A. 2005;102(43):15545–50. 16199517 10.1073/pnas.0506580102 PMC1239896

[27] Eden E, Navon R, Steinfeld I, Lipson D, Yakhini Z. GOrilla: a tool for discovery and visualization of enriched GO terms in ranked gene lists. BMC Bioinformatics. 2009;10(1):48. 19192299 10.1186/1471-2105-10-48 PMC2644678

[28] Supek F, Bošnjak M, Škunca N, Šmuc T. REVIGO summarizes and visualizes long lists of gene ontology terms. PLoS ONE. 2011;6(7):21800. 10.1371/journal.pone.0021800 PMC3138752 21789182

[29] Shannon P, Markiel A, Ozier O, Baliga NS, Wang JT, Ramage D, et al. Cytoscape: A software environment for integrated models of biomolecular interaction networks. Genome Res. 2003;13(11):2498–504. 14597658 10.1101/gr.1239303 PMC403769

[30] Xu S, Hu E, Cai Y, Xie Z, Luo X, Zhan L, et al. Using clusterProfiler to characterize multiomics data. Nat Protoc. 2024;19(11):3292–320. 39019974 10.1038/s41596-024-01020-z

[31] Sasaki T, Shiohama A, Minoshima S, Shimizu N. Identification of eight members of the Argonaute family in the human genome. Genomics. 2003;82(3):323–30. 12906857 10.1016/s0888-7543(03)00129-0

[32] Weng W, Li H, Goel A. Piwi-interacting RNAs (piRNAs) and cancer: emerging biological concepts and potential clinical implications. Biochim Biophys Acta Rev Cancer. 2019;1871(1):160–9. 30599187 10.1016/j.bbcan.2018.12.005 PMC6392428

[33] Hu W, Sun X, Ye T, Feng S, Ruan Q, Xi M, et al. PIWIL2 may serve as a prognostic predictor in cancers: a systematic review and meta-analysis. J BUON. 2021;25(6):2721–30. 33455119

[34] Li J, Xu L, Bao Z, Xu P, Chang H, Wu J, et al. High expression of PIWIL2 promotes tumor cell proliferation, migration and predicts a poor prognosis in glioma. Oncol Rep. 2017;38(1):183–92. 28534979 10.3892/or.2017.5647

[35] Feng D, Yan K, Zhou Y, Liang H, Liang J, Zhao W, et al. Piwil2 is reactivated by HPV oncoproteins and initiates cell reprogramming via epigenetic regulation during cervical cancer tumorigenesis. Oncotarget. 2016;7(40):64575–88. 27602489 10.18632/oncotarget.11810 PMC5323100

[36] Li Y, Wang W, Xu D, Liang H, Yu H, Zhou Y et al. PIWIL2/PDK1 axis promotes the progression of cervical epithelial lesions via metabolic reprogramming to maintain tumor-initiating cell stemness. Adv Sci (Weinh). 2024;2410756. 10.1002/advs.202410756 PMC11672288 39499767

[37] Qu X, Liu J, Zhong X, Li X, Zhang Q. PIWIL2 promotes progression of non-small cell lung cancer by inducing CDK2 and cyclin A expression. J Transl Med. 2015;13;301. 10.1186/s12967-015-0666-y PMC4571108 26373553

[38] Wang QE, Han C, Milum K, Wani AA. Stem cell protein Piwil2 modulates chromatin modifications upon cisplatin treatment. Mutat Res. 2011;708(1–2):59–68. 21310163 10.1016/j.mrfmmm.2011.02.001 PMC3091508

[39] Dinh NTM, Nguyen TM, Park MK, Lee CH, Y-Box Binding. Protein 1: unraveling the multifaceted role in cancer development and therapeutic potential. Int J Mol Sci. 2024;25(2):717. 38255791 10.3390/ijms25020717 PMC10815159

[40] Feng M, Xie X, Han G, Zhang T, Li Y, Li Y, et al. YBX1 is required for maintaining myeloid leukemia cell survival by regulating BCL2 stability in an m6A-dependent manner. Blood. 2021;138(1):71–85. 33763698 10.1182/blood.2020009676 PMC8667054

[41] Watanabe T, Lin H. Posttranscriptional regulation of gene expression by piwi proteins and piRNAs. Mol Cell. 2014;56(1):18–27. 25280102 10.1016/j.molcel.2014.09.012 PMC4185416

[42] Lee HE, Ayarpadikannan S, Kim HS. Role of transposable elements in genomic rearrangement, evolution, gene regulation and epigenetics in primates. Genes Genet Syst. 2016;90(5):245–57. 10.1266/ggs.15-00016 26781081

[43] Merkerova MD, Krejcik Z. Transposable elements and piwi-interacting RNAs in hemato-oncology with a focus on myelodysplastic syndrome (review). Int J Oncol. 2021;59(6):1–15. 10.3892/ijo.2021.5285 34779490

[44] Wu L, Xia M, Sun X, Han X, Zu Y, Jabbour EJ, et al. High levels of immunoglobulin expression predict shorter overall survival in patients with acute myeloid leukemia. Eur J Haematol. 2020;105(4):449–59. 32535947 10.1111/ejh.13466

[45] Kapitonov VV, Koonin EV. Evolution of the RAG1-RAG2 locus: both proteins came from the same transposon. Biol Direct. 2015;10(1):20. 25928409 10.1186/s13062-015-0055-8 PMC4411706

[46] Kaisrlikova M, Kundrat D, Koralkova P, Trsova I, Lenertova Z, Votavova H, et al. Attenuated cell cycle and DNA damage response transcriptome signatures and overrepresented cell adhesion processes imply accelerated progression in patients with lower-risk myelodysplastic neoplasms. Int J Cancer. 2024;154(9):1652–68. 38180088 10.1002/ijc.34834

[47] Farahani RK, Soleimanpour S, Golmohammadi M, Soleimanpour-Lichaei HR. PIWIL2 regulates the proliferation, apoptosis and colony formation of Colorectal Cancer Cell line. Iran J Biotechnol. 2023;21(1):36–44. 10.30498/ijb.2022.307054.3176 PMC9938935 36811102

[48] Leng X, Zhang M, Xu Y, Wang J, Ding N, Yu Y, et al. Non-coding RNAs as therapeutic targets in cancer and its clinical application. J Pharm Anal. 2024;14(7):100947. 39149142 10.1016/j.jpha.2024.02.001 PMC11325817

[49] To KKW, Zhang H, Cho WC. Competing endogenous RNAs (ceRNAs) and drug resistance to cancer therapy. Cancer Drug Resist. 2024;7;37. 10.20517/cdr.2024.66 PMC11472581 39403602

[50] Zou Y, Yang A, Chen B, Deng X, Xie J, Dai D, et al. crVDAC3 alleviates ferroptosis by impeding HSPB1 ubiquitination and confers trastuzumab deruxtecan resistance in HER2-low breast cancer. Drug Resist Updat. 2024;77:101126. 39243601 10.1016/j.drup.2024.101126

[51] Deng X, Liao T, Xie J, Kang D, He Y, Sun Y, et al. The burgeoning importance of PIWI-interacting RNAs in cancer progression. Sci China Life Sci. 2024;67(4):653–62. 38198029 10.1007/s11427-023-2491-7

[52] Mai D, Ding P, Tan L, Zhang J, Pan Z, Bai R, et al. PIWI-interacting RNA-54265 is oncogenic and a potential therapeutic target in colorectal adenocarcinoma. Theranostics. 2018;8(19):5213–230. 30555542 10.7150/thno.28001 PMC6276099

