# Supplementary material for: Dysregulation of transposable elements and PIWI-interacting RNAs in myelodysplastic neoplasms
Source: Biomark Res. 2025 Jan 23;13:13. doi: 10.1186/s40364-025-00725-x (PMC11755807; doi:10.1186/s40364-025-00725-x)
Supplement: Supplementary file 1 — Supplementary Material 1 [file 40364_2025_725_MOESM1_ESM.pdf]

Dysregulation of Transposable Elements and Piwi-Interacting RNAs in Myelodysplastic Neoplasms

Zdenek Krejcik, David Kundrat, Jiri Klema, Andrea Hrustincova, Iva Trsova, Monika Belickova, Jaroslav Cermak, Anna Jonasova, Michaela Dostalova Merkerova

SUPPLEMENTARY INFORMATION

SI Table 1. Characteristics of the cohort.

**Variable**	
Number of samples	97
Healthy controls	**17**
Sex (male/female)	8/9
Age, mean (range)	43 (27-71)
Patients	**80**
Sex (male/female)	40/40
Age, mean (range)	65 (27-83)
Diagnosis<br>(SLD/MLD/RS/5q-/EB1/EB2)	 7/18/11/12/11/21
IPSS-R category<br>(very low/low/intermediate/high/very high/n.a.*)	 17/26/15/12/9/1
Marrow blasts [%]: mean (range)	6.1 (0.0-19.6)
Hemoglobin (g/L): mean (range)	100 (51-149)
Neutrophils ($x10^9/L$): mean (range)	2.4 (0.2-14.5)
Platelets ($x10^9/L$): mean (range)	192 (10-597)
IPSS-R karyotype<br>(very good/good/intermediate/poor/very poor/n.a.)	 2/59/11/2/5/1
Cytogenetic features<br>normal karyotype<br>isolated del(5q)<br>isolated del(20q)<br>isolated +8<br>-Y<br>complex<br>other	 42<br>13<br>2<br>3<br>3<br>8<br>9
Somatic mutations<br>no. of mutations per patient: 0/1/2/3/4/5/6<br>splicing factors (*SF3B1*/*SRSF2*/*U2AF1*/*ZRSR2*)<br>epigenetic factors (*DNMT3A*/*TET2*/*ASXL1*)<br>*RUNX1*<br>*TP53*	 16/19/22/15/4/2/2<br>24/4/4/3<br>16/12/9<br>14<br>11
Follow-up, number of patients<br>mean follow-up (range) [months]	78<br>41 (0.5-153)
i. HSCT (censored), number of patients<br>mean time to HSCT (range) [months]	7<br>8 (0.5-23)
ii. progression, number of patients<br>mean time to progression (range) [months]	47<br>26 (0.3-100)
iii deceased, number of patients<br>mean time to death (range) [months]	45<br>32 (0.9-100)
iv. alive (censored), number of patients<br>mean follow-up time (range) [months]	33<br>52 (0.5-153)

n.a. – not analyzed, SLD – single lineage dysplasia, MLD – multilineage dysplasia, EB – excess of blasts, IPSS-R – revised international prognostic scoring system.

SI Table 2. Sequences of primers and probes used for RT–qPCR or ddPCR of individual piRNAs, TEs, and PCGs.

**FAM** (Custom Taqman Gene Expression assay)	Sequence
Forward Primer	AGGCTGTAGTGCGCTATGATC
Reverse Primer	GCTATGTTGCTCAGGCTGGA
Reporter	CCTGTGAATAGCCACTGCAC
**piR_018780** (Custom Taqman Small RNA assay)	Sequence
Context Sequence*	TTTCTGTGTGGAATTTGAATATCTGAAA
* sequence of RT-primer and PCR-primers not provided by the manufacturer	
PCGs	Assay ID
PIWIL2 (Taqman Gene Expression assay)	Hs01032720_m1
HPRT1 (Taqman Gene Expression assay)	Hs02800695_m1

SI Figure 1. A schematic flowchart summarizing methodological approach.

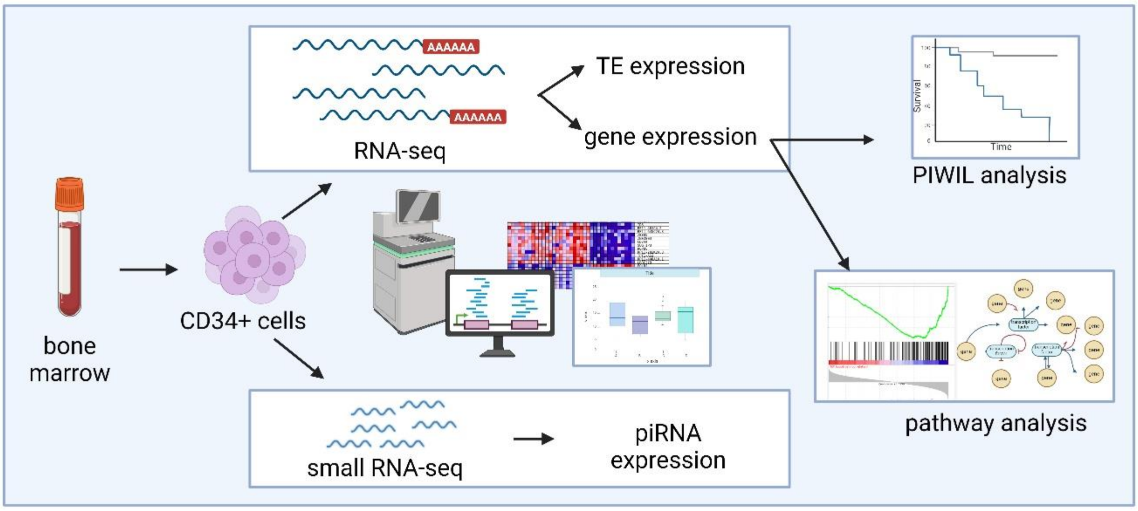

SI Figure 2. DEA comparison of expression of transposable elements (TEs) between samples from MDS patients and controls (CTR). (A) Heatmap of 13 significantly dysregulated TEs. Red indicates upregulation, blue indicates downregulation of TE expression, and the color intensity indicates the level of differential expression. The columns in the heatmap represent individual samples. (B) Boxplots of normalized counts from RNA-seq data of the three most significantly dysregulated TEs between MDS patients and controls. *** FDR < 0.001, ** FDR < 0.01.

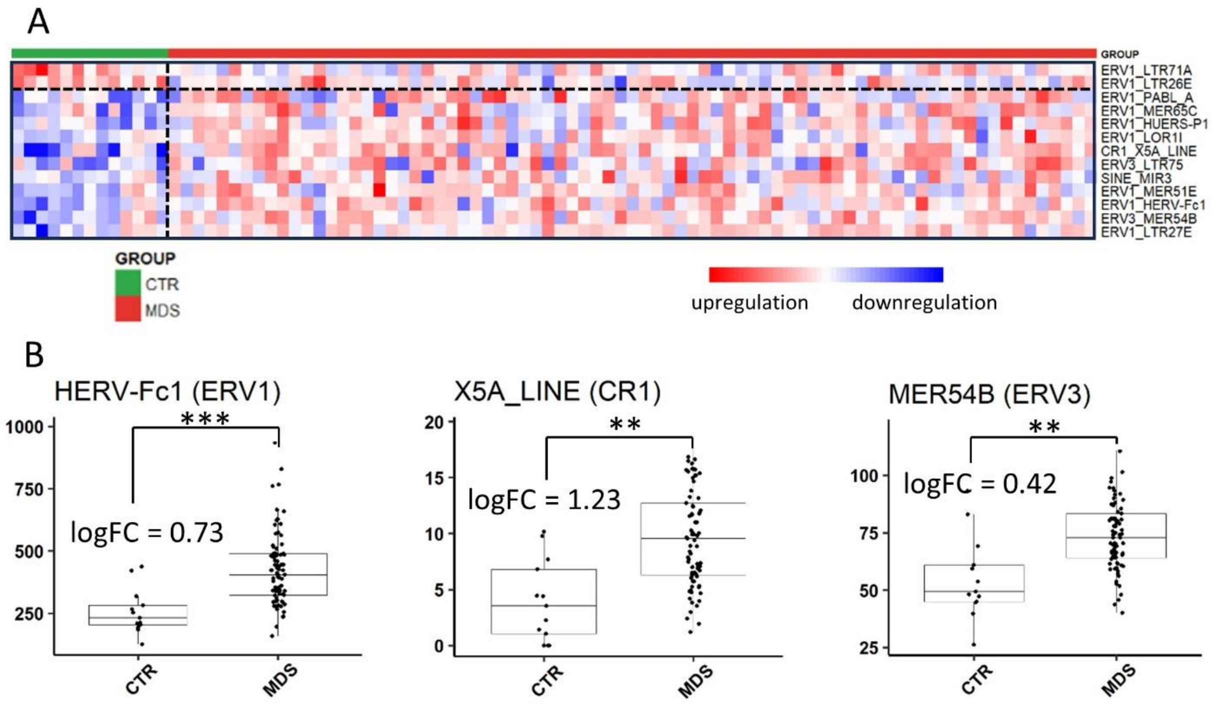

SI Table 3. Significantly dysregulated transposable elements (TEs) in DEA between MDS patients and controls (CTR). Normalized counts from RNA-seq outputs for both groups of samples are included in the CTR and MDS columns. FC – fold change, FDR – false discovery rate.

class	clade	TE	CTR	MDS	FC	log2(FC)	FDR
LTR Retrotransposon	ERV1	HERV-Fc1	257	425	1.65	0.73	0.0002
Non-LTR Retrotransposon	CR1	X5A_LINE	4	10	2.34	1.23	0.0014
LTR Retrotransposon	ERV3	MER54B	55	74	1.34	0.42	0.0072
LTR Retrotransposon	ERV1	LTR71A	307	250	0.81	-0.30	0.0130
LTR Retrotransposon	ERV1	MER51E	114	146	1.28	0.35	0.0130
LTR Retrotransposon	ERV1	LTR27E	34	49	1.42	0.51	0.0130
LTR Retrotransposon	ERV1	HUERS-P1	1505	1683	1.12	0.16	0.0308
LTR Retrotransposon	ERV1	LTR26E	79	58	0.73	-0.45	0.0358
LTR Retrotransposon	ERV1	PABL_A	816	901	1.11	0.14	0.0358
Non-LTR Retrotransposon	SINE	MIR3	21	49	2.28	1.19	0.0358
LTR Retrotransposon	ERV1	MER65C	34	44	1.31	0.39	0.0363
LTR Retrotransposon	ERV3	LTR75	2	4	2.24	1.16	0.0363
LTR Retrotransposon	ERV1	LOR1I	888	1026	1.16	0.21	0.0490

SI Table 4. The most significantly dysregulated transposable elements (TEs) in DEA between LR- and HR-MDS patients. Because 106 TEs were significantly dysregulated at an FDR < 0.05, the cutoff value for this DEA analysis was changed to an FDR < 0.001. Normalized counts from RNA-seq outputs for both groups of samples are included in the LR and HR columns. FC – fold change, FDR – false discovery rate.

class	clade	TE	LR	HR	FC	log2(FC)	FDR
Non-LTR Retrotransposon	SINE	AluYb8	8076	5776	0.72	-0.48	7.6E-06
LTR Retrotransposon	ERV1	LTR24C	95	119	1.25	0.33	2.0E-05
Non-LTR Retrotransposon	SINE	AluYe2	588	391	0.66	-0.59	3.1E-05
Non-LTR Retrotransposon	SINE	AluYb3a2	2967	2236	0.75	-0.41	5.0E-05
Non-LTR Retrotransposon	SINE	AluY	22750	16524	0.73	-0.46	6.9E-05
Non-LTR Retrotransposon	SINE	AluYd8	1481	931	0.63	-0.67	8.9E-05
Non-LTR Retrotransposon	SINE	AluYe5	6356	5058	0.80	-0.33	1.5E-04
Non-LTR Retrotransposon	SINE	AluYa1	1430	1086	0.76	-0.40	2.3E-04
LTR Retrotransposon	ERV1	LTR12E	3408	2646	0.78	-0.37	3.6E-04
LTR Retrotransposon	ERV1	HARLEQUIN	5705	7953	1.39	0.48	3.6E-04
Non-LTR Retrotransposon	SINE	AluYc1	3396	2615	0.77	-0.38	3.9E-04
LTR Retrotransposon	ERV1	MER52A	417	356	0.85	-0.23	6.3E-04
Non-LTR Retrotransposon	SINE	AluSq4	7871	6672	0.85	-0.24	8.4E-04
LTR Retrotransposon	ERV1	PABL_AI	1526	1772	1.16	0.22	8.4E-04

SI Figure 3. Boxplots of normalized counts from RNA-seq data for three piRNAs whose expression was significantly dysregulated between MDS patients and controls (CTR). ** FDR < 0.01.

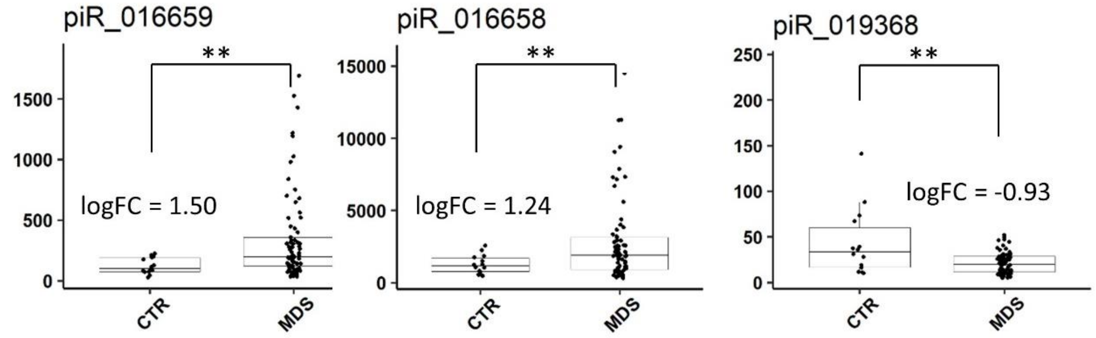

SI Table 5. Significantly dysregulated piRNAs in DEA between LR- and HR-MDS patients. Normalized counts from small RNA-seq outputs for both groups of samples are included in the LR and HR columns. FC – fold change, FDR – false discovery rate.

piRNA	LR	HR	FC	log2(FC)	FDR
piR_019521	17	1	0.077	-3.71	0.0005
piR_020009	211	98	0.463	-1.11	0.0019
piR_020814	41	16	0.395	-1.34	0.0020
piR_009051	16	3	0.299	-1.74	0.0025
piR_019420	12	5	0.375	-1.41	0.0036
piR_005271	107	165	1.531	0.61	0.0070
piR_017791	184	297	1.602	0.68	0.0070
piR_020813	371	584	1.576	0.66	0.0134
piR_020381	11	6	0.399	-1.33	0.0173
piR_001152	672	946	1.400	0.49	0.0173
piR_017458	5542	8682	1.567	0.65	0.0194
piR_000586	189	303	1.602	0.68	0.0194
piR_009228	35	16	0.465	-1.10	0.0198
piR_020657	22	36	1.618	0.69	0.0215
piR_020829	485	304	0.627	-0.67	0.0376
piR_017033	83	60	0.732	-0.45	0.0418
piR_020450	676	444	0.658	-0.60	0.0430

SI Figure 4. Total transposable element (TE) and piRNA levels in MDS patients stratified according to (A) the WHO diagnostic criteria and (B) the IPSS-R risk score. Normalized counts from RNA-seq (TEs) and small RNA-seq (piRNAs) data are shown. Welch's ANOVA was used to assess the statistical significance of differences between groups of samples.

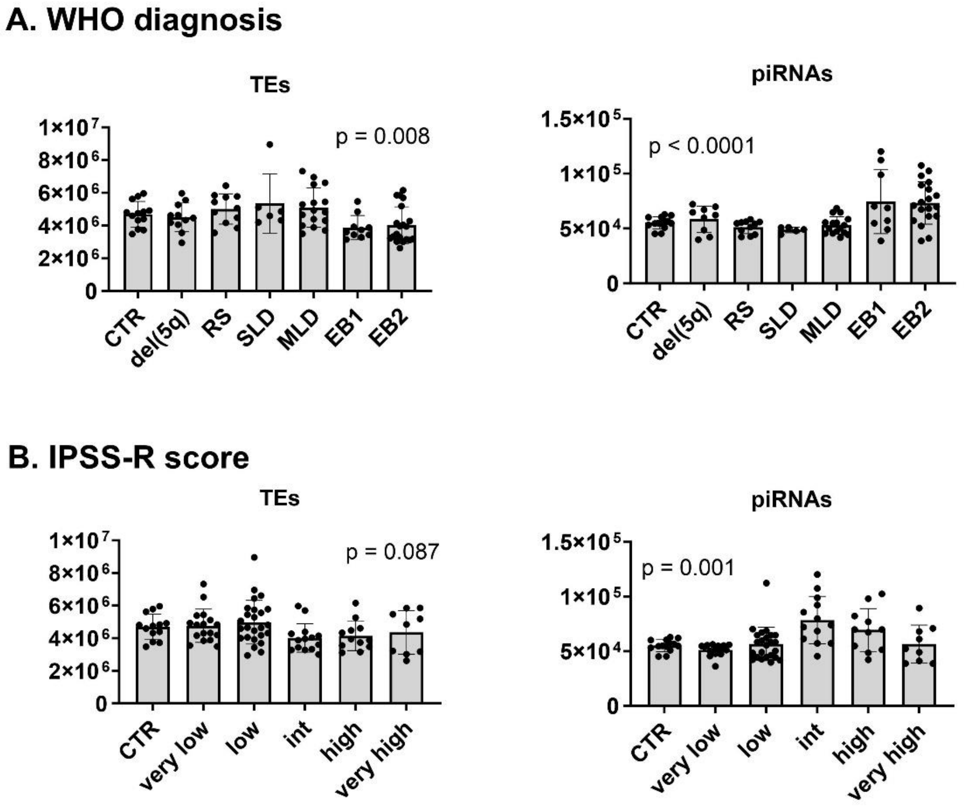

SI Table 6. Transposable elements (TEs) and piRNAs significantly dysregulated in DEA between MDS patients with del5q and those with a normal karyotype (n.k.). Normalized counts for both groups of samples are included in the n.k. and del5q columns. FC – fold change, FDR – false discovery rate.

class	clade	GENE	n.k.	del5q	FC	log2(FC)	FDR
Non-LTR Retrotransposon	L1	L1MB5	1545	1791	1.16	0.21	0.0027
LTR Retrotransposon	ERV1	LTR10C	2266	2598	1.15	0.20	0.0027
LTR Retrotransposon	ERV1	LTR1B0	84	48	0.58	-0.79	0.0027
LTR Retrotransposon	ERV1	MER61B	88	69	0.78	-0.36	0.0027
Non-LTR Retrotransposon	SINE	MIR	470	587	1.25	0.32	0.0027
LTR Retrotransposon	ERV2	HERVK	3093	2303	0.74	-0.43	0.0031
LTR Retrotransposon	ERV1	MER4D	873	1057	1.21	0.28	0.0067
LTR Retrotransposon	ERV1	MER92C	8	14	1.78	0.83	0.0137
Non-LTR Retrotransposon	L1	L1M2A_5	209	249	1.19	0.26	0.0143
Non-LTR Retrotransposon	L1	L1MB2	2588	2927	1.13	0.18	0.0143
LTR Retrotransposon	ERV3	MLT1C2	686	754	1.10	0.14	0.0143
LTR Retrotransposon	ERV1	LTR43	141	111	0.79	-0.34	0.0192
Non-LTR Retrotransposon	L1	L1MA8	999	1123	1.12	0.17	0.0197
Non-LTR Retrotransposon	L1	L1MEC_5	349	403	1.16	0.21	0.0197
LTR Retrotransposon	ERV1	LTR10D	403	474	1.18	0.24	0.0197
LTR Retrotransposon	ERV1	LTR10G	221	260	1.18	0.24	0.0197
LTR Retrotransposon	ERV1	LTR1B1	113	86	0.76	-0.39	0.0197
LTR Retrotransposon	ERV1	LTR49	278	317	1.14	0.19	0.0197
LTR Retrotransposon	ERV1	LTR6A	220	282	1.28	0.35	0.0197
LTR Retrotransposon	ERV1	MER4A1	2308	2113	0.92	-0.13	0.0197
Non-LTR Retrotransposon	L1	L1MC3	1078	1229	1.14	0.19	0.0204
LTR Retrotransposon	ERV1	LTR38A1	15	10	0.64	-0.65	0.0204
Non-LTR Retrotransposon	SINE	AluYe2	580	384	0.66	-0.59	0.0210
LTR Retrotransposon	ERV2	HERV-K14CI	76	36	0.48	-1.07	0.0210
Non-LTR Retrotransposon	L1	L1ME3	240	293	1.22	0.29	0.0210
LTR Retrotransposon	ERV1	LTR1B	242	190	0.78	-0.35	0.0210
Non-LTR Retrotransposon	L1	L1PBB_5	109	87	0.80	-0.31	0.0210
Non-LTR Retrotransposon	L1	MER25	231	198	0.85	-0.23	0.0210
LTR Retrotransposon	ERV1	MER4CL34	106	134	1.26	0.33	0.0210
LTR Retrotransposon	ERV3	MER76-int	6	3	0.46	-1.13	0.0210
LTR Retrotransposon	ERV2	HERVK11DI	142	97	0.68	-0.55	0.0214
Non-LTR Retrotransposon	L1	L1MA10	425	488	1.15	0.20	0.0214
Non-LTR Retrotransposon	L1	L1MC2	1617	1817	1.12	0.17	0.0214
LTR Retrotransposon	ERV1	LTR65	35	50	1.42	0.50	0.0214
LTR Retrotransposon	ERV1	LTR45C	102	120	1.18	0.24	0.0256
Non-LTR Retrotransposon	L1	L1MB8	2292	2605	1.14	0.18	0.0304
LTR Retrotransposon	ERV1	LTR1	160	129	0.81	-0.31	0.0304
Non-LTR Retrotransposon	L1	L1MB1	1035	1127	1.09	0.12	0.0328
Non-LTR Retrotransposon	L1	L1MC1	4179	4635	1.11	0.15	0.0358
LTR Retrotransposon	ERV1	MER52C	423	365	0.86	-0.21	0.0376
LTR Retrotransposon	ERV2	MER9B	146	180	1.23	0.30	0.0387
Non-LTR Retrotransposon	CR1	L3	5	8	1.65	0.72	0.0414
LTR Retrotransposon	ERV1	MER51C	297	253	0.85	-0.23	0.0414
Non-LTR Retrotransposon	L1	L1MB4	2754	3068	1.11	0.16	0.0453

GENE	n.k.	del5q	FC	log2(FC)	FDR
piR_016658	1796	5165	2.88	1.52	0.0013
piR_016659	216	502	2.32	1.22	0.0251
piR_019050	41	25	0.62	-0.69	0.0454

SI Table 7. Transposable elements (TEs) and piRNAs significantly dysregulated between MDS patients with no mutation (wild type; wt) and those with a mutation in the *SF3B1* gene. Normalized counts for both groups of samples are included in the wt and *SF3B1* columns. FC – fold change, FDR – false discovery rate.

class	clade	GENE	wt	SF3B1	FC	log2(FC)	FDR
Non-LTR Retrotransposon	SINE	FRAM	2829	1864	0.66	-0.60	0.0085
LTR Retrotransposon	ERV1	HERV-Fc1	351	500	1.42	0.51	0.0085
LTR Retrotransposon	ERV1	LTR36	101	75	0.75	-0.42	0.0178
LTR Retrotransposon	ERV1	MER101_I	344	263	0.77	-0.38	0.0178
LTR Retrotransposon	ERV2	MER9a1	589	667	1.13	0.18	0.0178
Non-LTR Retrotransposon	SINE	AluYb9	4154	5338	1.28	0.36	0.0245

GENE	wt	SF3B1	FC	log2(FC)	FDR
piR_019521	2	21	10.77	3.43	0.0236
piR_019368	36	12	0.37	-1.45	0.0236
piR_020814	45	14	0.32	-1.66	0.0396

SI Figure 5. Validation of the outputs of NGS on the FAM transposable element and piR_018780 via RT–qPCR. RT–qPCR was performed on a subset of samples (15 MDS patients and 5 controls), and the levels of targets were normalized to those of the *HPRT1* reference gene control.

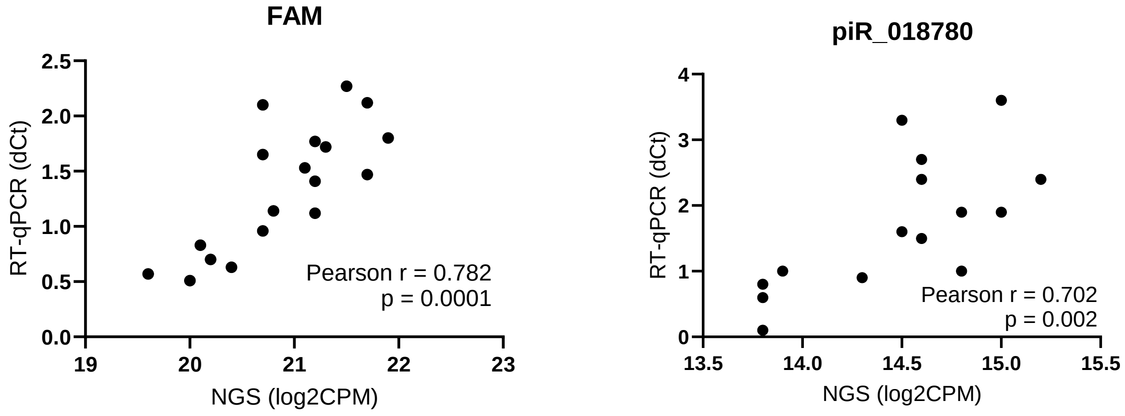

SI Figure 6. *PIWIL4* expression in MDS. (A) Differences in *PIWIL4* expression in the CTR, LR-MDS, and HR-MDS groups as defined by the IPSS-R. Normalized counts from the RNA-seq data are shown. Student´s t test was used to assess statistical significance between LR- and HR-MDS, ns – nonsignificant. (B) Correlation between *PIWIL4* expression and overall piRNA levels. Pearson's test was performed to evaluate the correlation. (C) Survival of MDS patients in relation to the *PIWIL4* level. Overall survival (OS) and progression-free survival (PFS) curves were plotted via the Kaplan–Meier method. Patients were divided into separate "low" and "high" groups on the basis of *PIWIL4* levels measured in CTR samples. The cutoff value was set as the maximum value detected in the CTR group, as indicated in (A). Differences between the curves were compared via the log-rank test. HR – hazard ratio, CI – confidence interval.

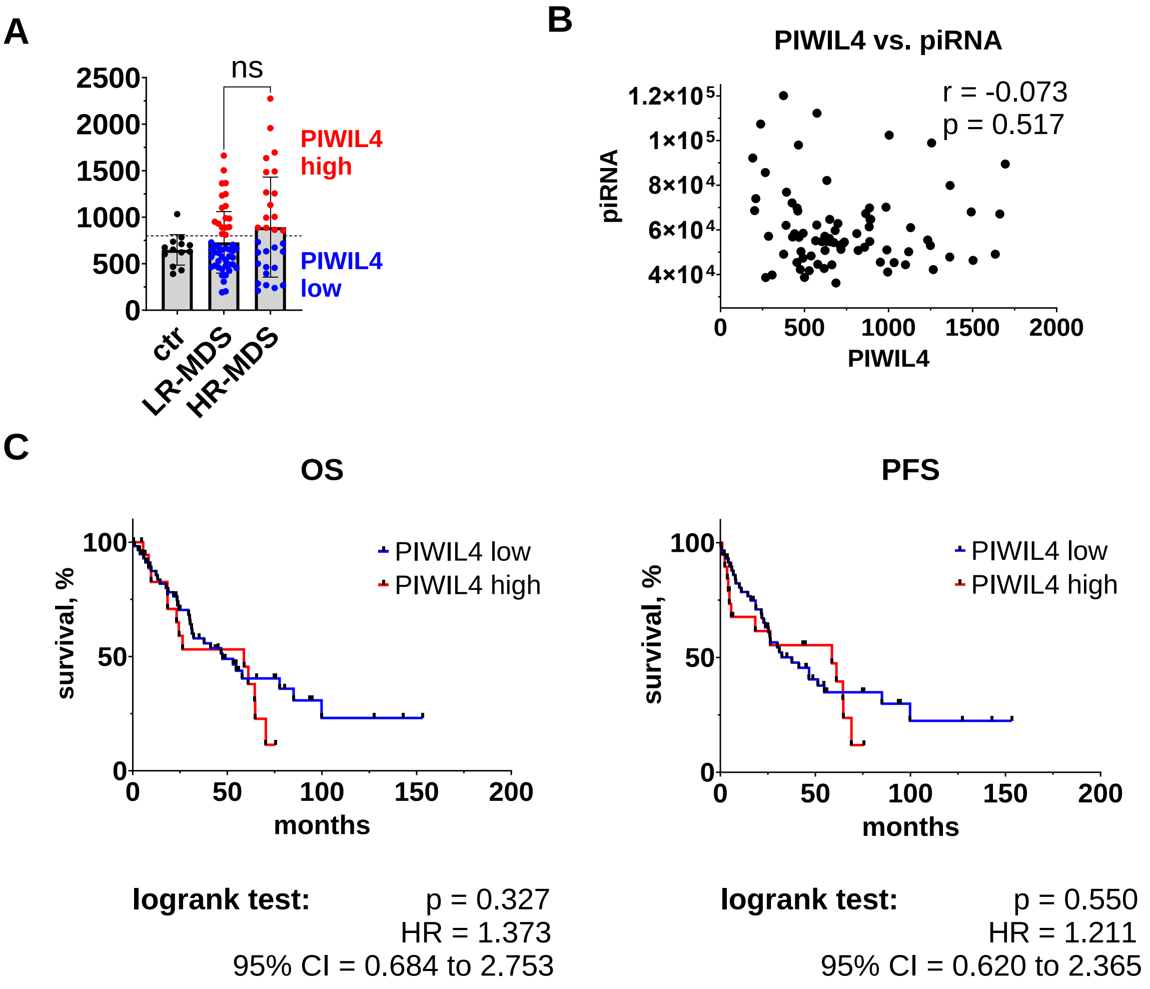

SI Figure 7. Validation of the association of the *PIWIL2* level with patient survival in an independent cohort of 72 MDS patients (eshtablished by Szikszai K, et al. *Cancers*. 2020;12(10):1–21.). Overall survival (OS) and progression-free survival (PFS) curves were plotted via the Kaplan–Meier method. Patients were divided into separate “low” and “high” groups with the maximum *PIWIL2* level observed in in CTR samples as the cutoff. Differences between the curves were compared via the log-rank test. HR – hazard ratio, CI – confidence interval.

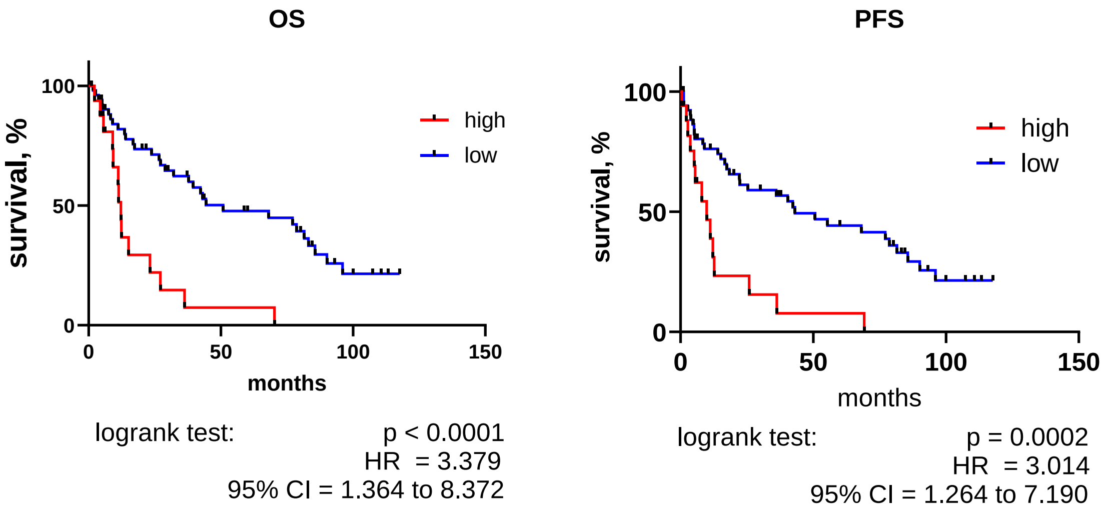

SI Figure 8. Relationship of *PIWIL2* expression and bone marrow (BM) blast count. Normalized counts of *PIWIL2* from RNA-seq are shown. Welch's ANOVA was used to assess the statistical significance of differences between groups of samples.

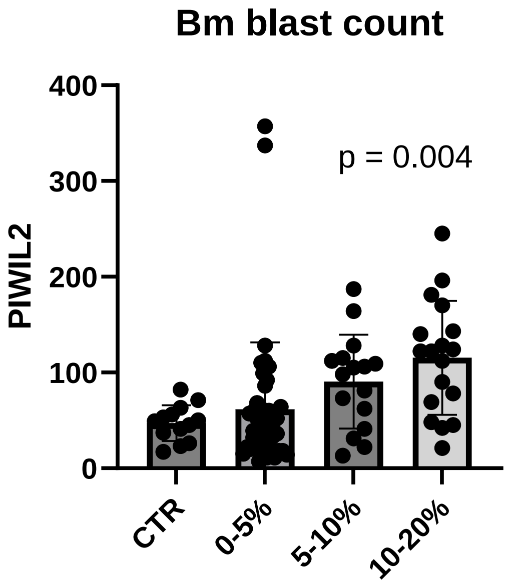

SI Figure 9. Gene network graphs and pathway enrichment data generated for (A) piR_018780, (B) FAM, and (C) HERV-Fc1. Three graphs composed of PCGs, TEs, and piRNAs were computationally modeled via RNA-seq and small RNA-seq data on the basis of pairwise correlations. The enrichment analysis was based on annotations of PCGs included in each graph, and significant GO terms are listed in the corresponding tables (adjusted p < 0.05).

A. piR_018780

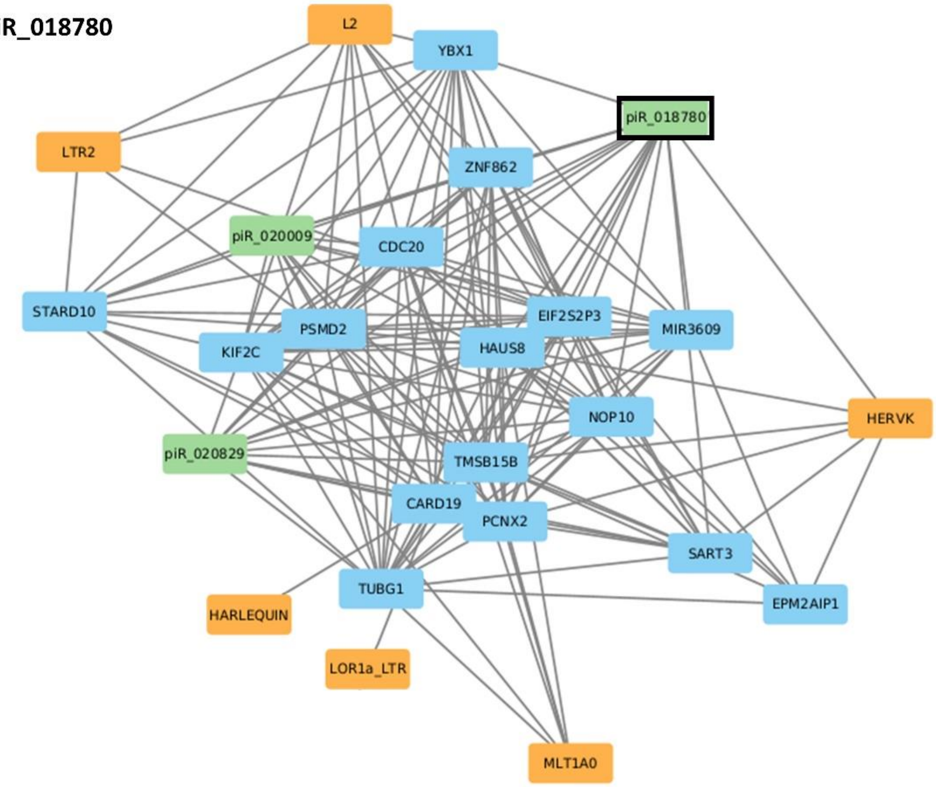

ID	Description	Genes	p.adjust
GO:0005819	spindle	CDC20, HAUS8, TUBG1, KIF2C	0,005
GO:0000793	condensed chromosome	TUBG1	0,014
GO:0015030	Cajal body	YBX1	0,015
GO:0007059	chromosome segregation	TUBG1	0,026
GO:0031109	microtubule polymerization or depolymerization	CDC20, KIF2C	0,026
GO:0000776	kinetochore	CDC20, HAUS8, TUBG1, KIF2C	0,036
GO:1990124	messenger ribonucleoprotein complex	NOP10	0,036
GO:0051258	protein polymerization	HAUS8, TUBG1, TMSB15B	0,038
GO:0000930	gamma-tubulin complex	HAUS8, TUBG1, KIF2C	0,044
GO:0000242	pericentriolar material	CDC20	0,044
GO:0005680	anaphase-promoting complex	CDC20	0,044
GO:0005697	telomerase holoenzyme complex	YBX1	0,044
GO:0005838	proteasome regulatory particle	PSMD2	0,044
GO:0098554	cytoplasmic side of endoplasmic reticulum membrane	EPM2AIP1	0,044
GO:0005732	sno(s)RNA-containing ribonucleoprotein complex	NOP10	0,049
GO:0005689	U12-type spliceosomal complex	SART3, NOP10	0,049

B. FAM

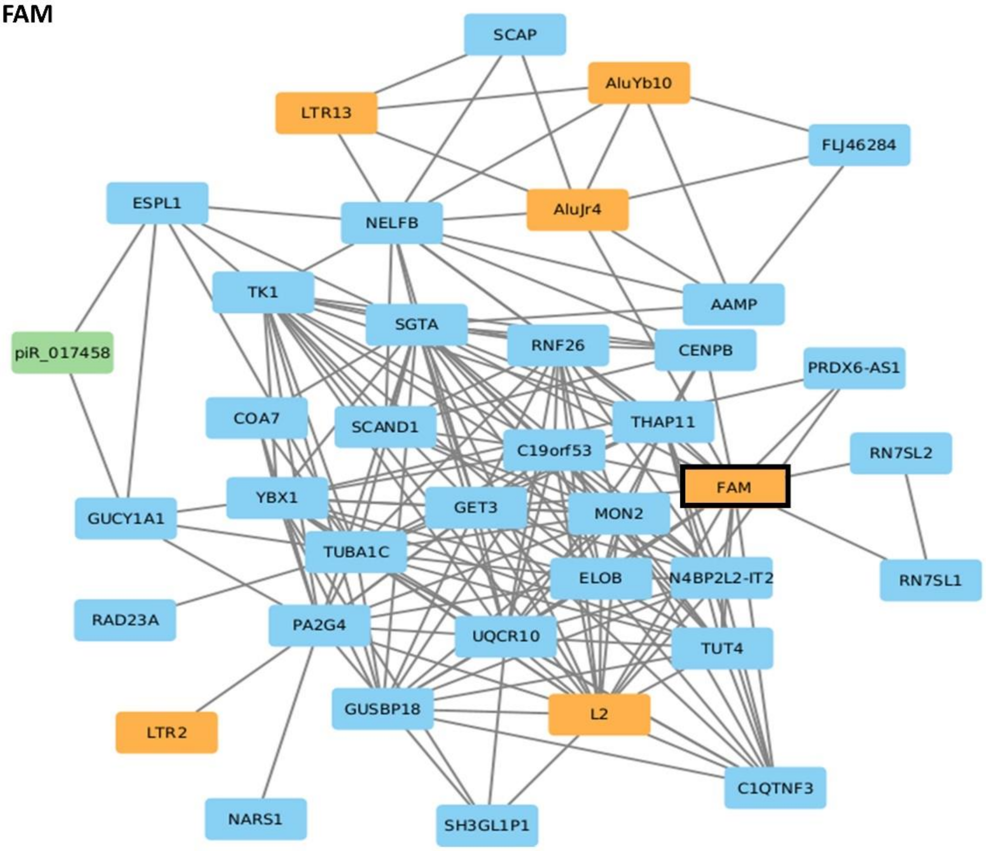

ID	Description	Genes	p.adjust
GO:0008023	transcription elongation factor complex	ELOB, NELFB	0,006
GO:0000153	cytoplasmic ubiquitin ligase complex	ELOB	0,028
GO:1990124	messenger ribonucleoprotein complex	YBX1	0,028
GO:0005786	signal recognition particle, endoplasmic reticulum targeting	RN7SL1	0,028
GO:0005689	U12-type spliceosomal complex	YBX1	0,038

C. HERV-Fc1

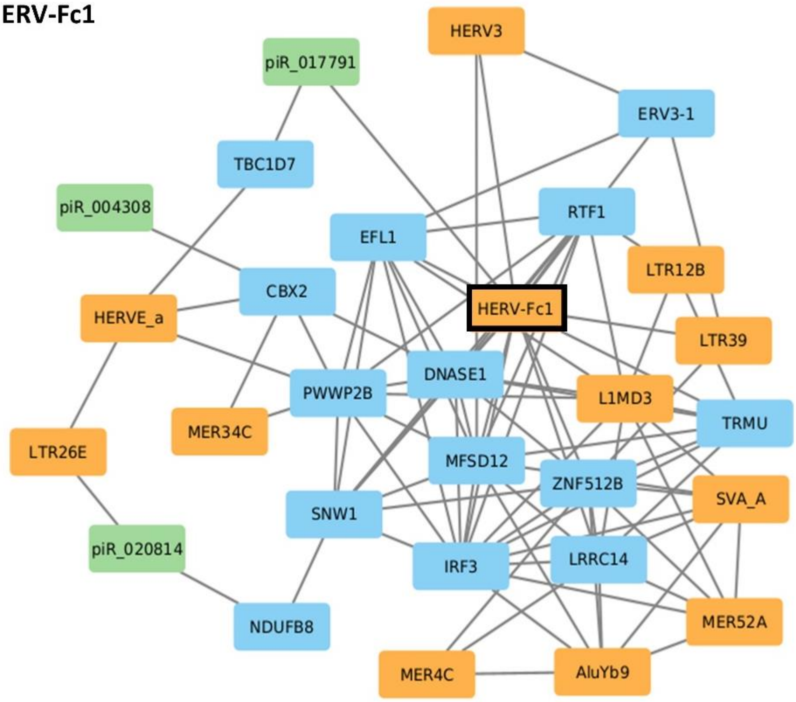

ID	Description	Genes	p.adjust
GO:0008023	transcription elongation factor complex	SNW1, RTF1	0,028
GO:1902018	negative regulation of cilium assembly	TBC1D7	0,038
GO:1904262	negative regulation of TORC1 signaling	TBC1D7	0,046
GO:1902116	negative regulation of organelle assembly	TBC1D7	0,046
GO:0031669	cellular response to nutrient levels	SNW1,TBC1D7	0,046
